# Exploring the Role of the Nephelauxetic Effect in Circularly Polarized Luminescence of Chiral Chromium(III) Complexes

**DOI:** 10.1021/jacs.5c06196

**Published:** 2025-06-28

**Authors:** Maxime Poncet, Laura Cuevas-Contreras, Yating Ye, Laure Guénée, Carlos M. Cruz, Claude Piguet, Juan-Ramón Jiménez

**Affiliations:** † Department of Inorganic and Analytical Chemistry, University of Geneva, Quai E. Ansermet 30, CH-1211 Geneva 4, Switzerland; ‡ Departamento de Química Inorgánica, Facultad de Ciencias, Unidad de Excelencia de Química Aplicada a Biomedicina y Medioambiente, Avda. Fuente Nueva s/n, 18071 Granada, Spain; § Laboratory of Crystallography, University of Geneva, Quai E. Ansermet 24, CH-1211 Geneva 4, Switzerland; ∥ Departamento de Química Orgánica, Facultad de Ciencias, Unidad de Excelencia de Química Aplicada a Biomedicina y Medioambiente, Avda. Fuente Nueva s/n, 18071 Granada, Spain

## Abstract

A novel chiral chromium­(III) molecular ruby [Cr­(qpp)_2_]^3+^ (qpp = *N*-methyl-*N*-(pyridin-2-yl)-6-(quinolin-8-yl)­pyridin-2-amine) has been synthesized, enantiomerically resolved, and fully characterized. The circularly polarized luminescence (CPL) spectra revealed two emission bands of opposite polarization in the near-infrared region (700–800 nm), corresponding to the metal-centered transitions Cr­(^2^T­(1) → ^4^A_2_) and Cr­(^2^E­(1) → ^4^A_2_). Notably, the dissymmetry factor *g*_lum_ reached 0.11 for the former transition, which is among the highest reported for chromium­(III) systems. Comparison with structurally related homo- and heteroleptic chromium­(III) complexes underscores the important role of the nephelauxetic effect in tuning CPL properties. Increased metal–ligand covalency, indicative of a stronger nephelauxetic effect, enhances orbital mixing and modifies the electronic character of the emissive states. These changes influence both electric and magnetic transition dipole moments, leading to noticeable variations in dissymmetry factor *g*_lum_. Altogether, these observations highlight the potential of fine-tuning metal–ligand covalency as a rational strategy for optimizing the chiroptical properties of chromium­(III) complexes, with promising implications for bioimaging, molecular probes, and circularly polarized optoelectronic devices.

## Graphical abstract

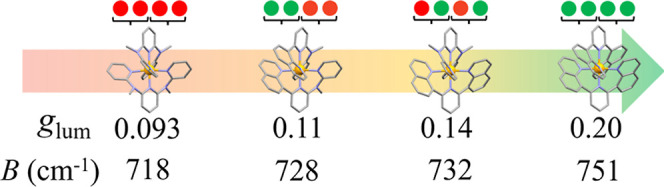

## Introduction

Chiral chromium­(III) complexes have garnered increasing interest as chiral luminophores due to their ability to exhibit a strong emission dissymmetry factor (*g*_lum_) and circularly polarized luminescence (CPL) brightness (*B*_CPL_).^1–4^ These appealing properties make them promising candidates for applications in bioimaging, light-emitting devices, and molecular probes.^5–8^ The sharp light emissions arising from the low-lying excited states Cr­(^2^E,^2^T_1_ → ^4^A_2_), so-called “spin-flip” transitions (SF), display long excited state lifetimes accompanied by high photoluminescence quantum yields (Φ_PL_) when strong field tridentate six-membered chelate ring ligands are coordinated to the metal center.^9,10^ As a result of the chemical inertness of chromium­(III), stable configuration and chirality can arise from the formation of the chelate rings with the helically twisted *C*_2_-symmetrical ditridentate ligands dqp and ddpd in [Cr­(**L**)_2_]^3+^ complexes (**L** = dqp = 2,6-di­(quinolin-8-yl)­pyridine, **L** = ddpd = *N* ^2^,*N* ^6^-dimethyl-*N* ^2^,*N* ^6^-di­(pyridin-2-yl)­pyridine-2,6-diamine; Figure 1a,d).^11–13^ The SF transitions in chromium­(III) complexes, which consist in the rearrangement of the electrons within the t_2_(π) orbitals, are forbidden by both the spin and Laporte selection rule, leading to comparable magnitudes for the electric (μ) and magnetic (*m*) transition dipole moments.^14,15^ This is crucial for enhancing the dissymmetry factor (*g*_lum_), which represents the degree of “enantiorichness” of the CPL emitted by a chiral luminophore at a given wavelength (eq. 1). Moreover, to maximize the *g*_lum_, these two vectors should be collinear (θ = 0° or 180°).^1,16^

1 $$\left|g_{lum}\right|=4\times \frac{\left(\mu /m\right)\times \cos\;\theta }{{\left(\mu /m\right)}^{2}+1}$$

**Figure 1:**
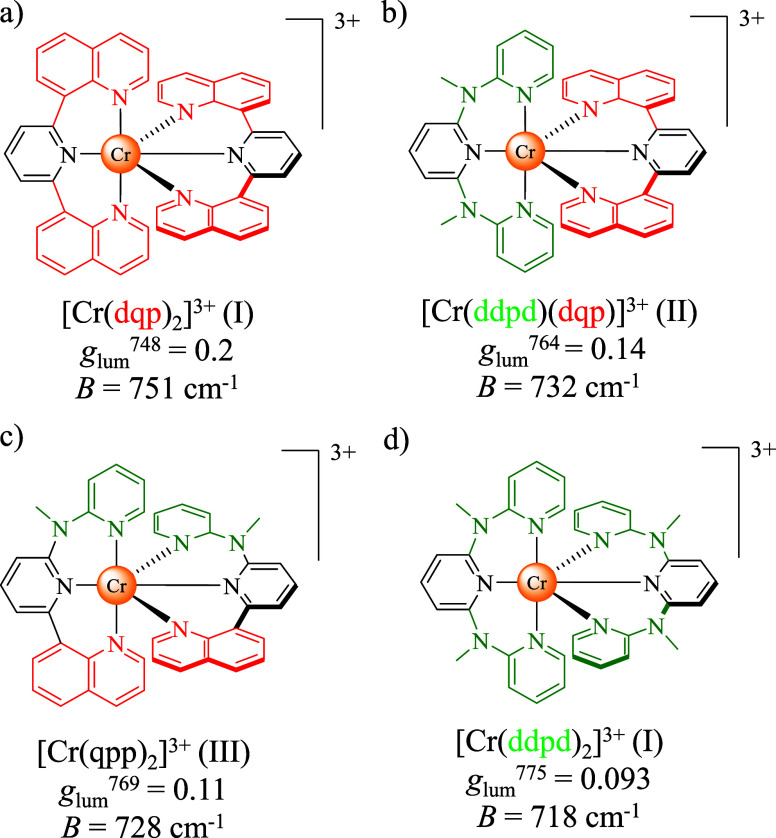
Molecular structures of (a) [Cr­(dqp)_2_]^3+^, (b) [Cr­(dqp)­(ddpd)]^3+^, (c) [Cr­(qpp)_2_]^3+^, and (d) [Cr­(ddpd)_2_]^3+^ with their respective *g*_lum_ and Racah parameter *B*.

Since the energies of SF states in this type of compounds are largely independent of the ligand-field splitting (Δ_oct_), increasing metal–ligand covalency through the nephelauxetic effect presents a viable strategy for shifting these states to lower energies.^17–23^ However, this approach comes at a cost as the enhanced covalency leads to increased mixing or “cloud-expanding” of the metal–ligand orbitals, resulting in a loss of their pure SF character and thus the modification of the transition dipole moments of the transition. This, in turn, can impact the magnitude of *g*_lum_.^24–26^ In this work, we investigate how subtle variations in the metal–ligand covalency (nephelauxetic effect) affect the magnitude of *g*_lum_ in a series of structurally related chiral chromium­(III) complexes (Figure 1).

## Results and Discussion

### Synthesis and Structural Properties

As a starting point, let us consider the previously studied helically chiral homoleptic *PP*/*MM*-[Cr­(dqp)_2_]^3+^ and *PP*/*MM*-[Cr­(ddpd)_2_]^3+^ (*D*_2_-symmetry, structure I in Scheme S1) and heteroleptic *PP*/*MM*-[Cr­(dqp)­(ddpd)]^3+^ (*C*_2_-symmetry, structure II in Scheme S1) compounds, the dissymmetry factors of which reach |*g*_lum_^748^| = 0.20, |*g*_lum_^775^| = 0.093 and |*g*_lum_^764^| = 0.14, respectively, for the lower energy SF transition (Figure 1a,b,d).^4,13,27^ The alternative side-by-side nonchiral *PM* isomers are destabilized by the lack of interstrand packing interactions and were never observed. They are therefore not considered further in this work. For exploring chirality beyond symmetrical double-helical arrangement in these systems, a new inert chromium complex [Cr­(qpp)_2_]^3+^ has been prepared using the dissymmetric ligand qpp (qpp = *N*-methyl-*N*-(pyridin-2-yl)-6-(quinolin-8-yl)­pyridin-2-amine, Figure 2 and Supporting Information for synthetic details) which corresponds to a “half-dqp/half-ddpd” tridentate binding unit containing a central pyridine flanked with a quinoline and a *N*-methylpyridin-2-amine terminal groups (Figure 1c). The homoleptic complex *rac*-[Cr­(qpp)_2_]^3+^ could be synthesized by reaction with labile Cr^II^(SO_3_CF_3_)_2_ followed by oxidation with AgSO_3_CF_3_ to yield air-stable inert *rac*-[Cr­(qpp)_2_]^3+^ (Figure 2 and Supporting Information for synthetic details).

**Figure 2:**
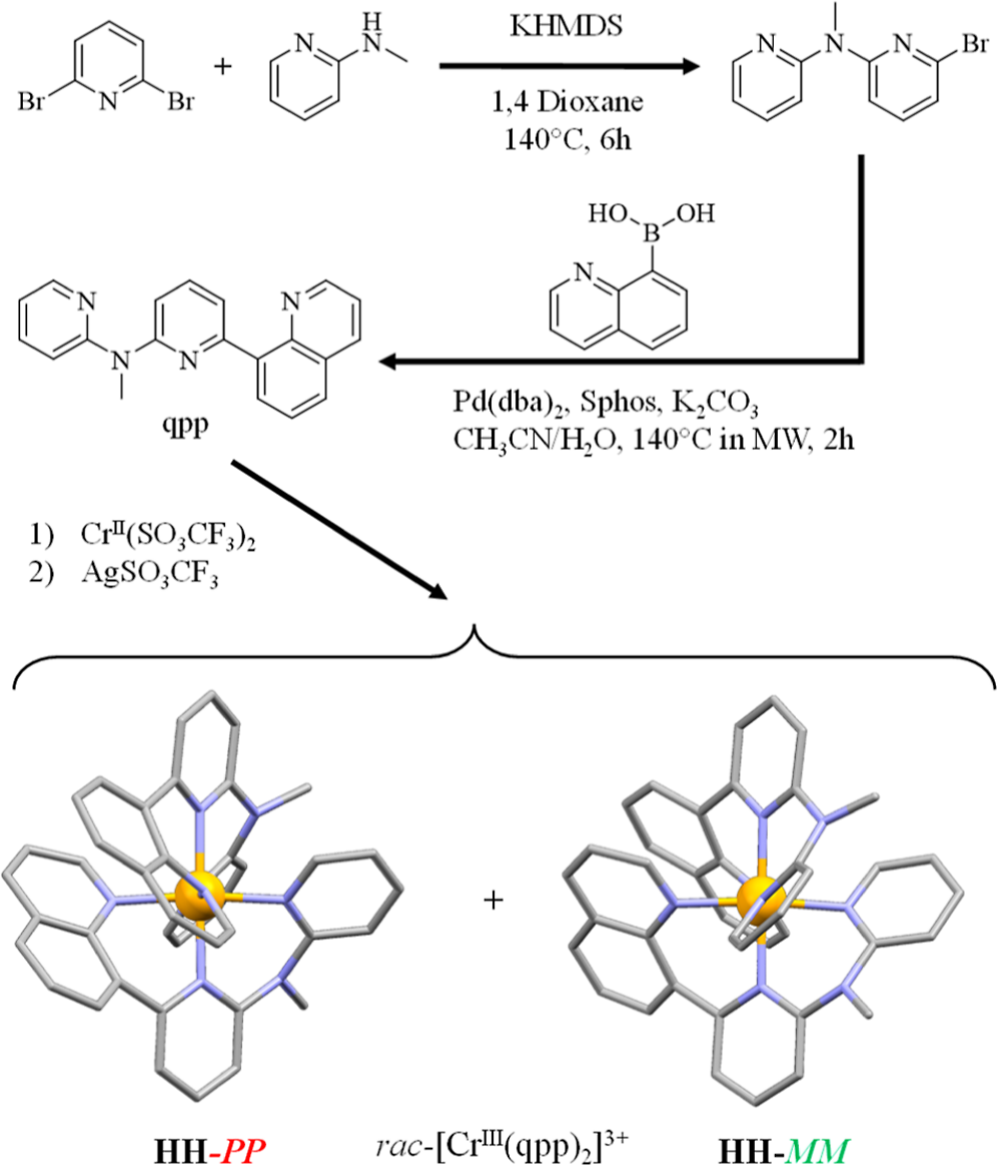
Synthesis of the organic ligand qpp and synthesis of the homoleptic complex *rac*-HH-[Cr­(qpp)_2_]­(SO_3_CF_3_)_3_.

Slow diffusion of diethyl ether in a concentrated solution of methanol yielded X-ray-quality crystals, showing the exclusive formation of a racemic mixture of the head-to-head HH-*PP*/*MM* [Cr­(qpp)_2_]^3+^ diastereomer (*C*_2_-symmetry, structure III in Scheme S1) where both ligands are meridionally wrapped around the metallic center (Figures 2 and S1 and Tables S1 and S2, CCDC-2428511), thus allowing the operation of stabilizing interstrand homotopic quinoline/quinoline interactions (interplanar angles of 15.53°, Figures 2 and S2 and S3). The transoid bite angles N–Cr–N are in the 173.76(14)°–178.71(16)° range, in line with only minor distortions from perfect octahedron ascertained by (i) Σ = ∑_*i*=1_^12^|90 – φ_*i*_| = 31.97° (φ_*i*_ are the cisoid bite angles N–Cr–N) similar to those reported for heteroleptic [Cr­(dqp)­(ddpd)]^3+^ and homoleptic [Cr­(dqp)_2_]^3+^ and [Cr­(ddpd)_2_]^3+^ parent complexes (28.9° to 37.1°, Figure S2) and (ii) continuous shape measurements (CShMs,^28^ Table S3). Thus, all four compounds exhibit essentially identical geometrical characteristics, both in their primary coordination spheres and in their ligand distortions. However, one notes that the average Cr–N bond distance in the “full-terminal quinoline” [Cr­(dqp)_2_]^3+^ complex (2.060(3) Å) is longer than those observed in all complexes containing terminal pyridine ligands in [Cr­(ddpd)_2_]^3+^ (2.044(5) Å), [Cr­(dqp)­(ddpd)]^3+^ (2.045(5) Å) and [Cr­(qpp)_2_]^3+^ (2.047(3) Å, Figure S2), which suggests variable covalent characters and different nephelauxetic effect (vide infra).

### Absorption and Emission Properties

The absorption spectrum of the novel HH-*rac*-[Cr­(qpp)_2_]^3+^compound was recorded at room temperature in acetonitrile at different concentrations to observe all of the active transitions of the complex (Figures 3 and S4 and Table S4). The 250–350 nm region (40,000–28,571 cm^–1^) is dominated by intense allowed ligand-centered π* ← π transitions. Charge transfers (CTs) transitions are located in the visible range 350–500 nm (28,571–20,000 cm^–1^) while the shoulder located at 420–440 nm (23,810–22,727 cm^–1^) has been tentatively assigned to the spin-allowed metal-centered (MC) transition Cr­(^4^T_2_ ← ^4^A_2_) according to TD-DFT calculations (Tables S8 and S10 and Figures S16, S19, S21 and S22). Because of the d^3^ electronic configuration in the octahedral geometry, this transition is a direct measure of the ligand field splitting energy Δ_oct_. Similar values of Δ_oct_ are found within the family (Table 1, column 4). At lower energy, the weak doubly forbidden SF transitions Cr­(^2^T_1_,^2^E ← ^4^A_2_) are observed within the 680–800 nm (14,706–12,500 cm^–1^) range with ε reaching 0.14 M^–1^ cm^–1^ (Figures 3 inset and S4 and Table S4). The (very) minor splitting of the chromium­(III)-based spin-flip transitions supports (Figures 3 and S18) the use of octahedral irreducible labels (^4^A_2_, ^2^E, ^2^T_1_, and ^4^T_2_) for characterizing the intrashell d–d transitions.

**Figure 3:**
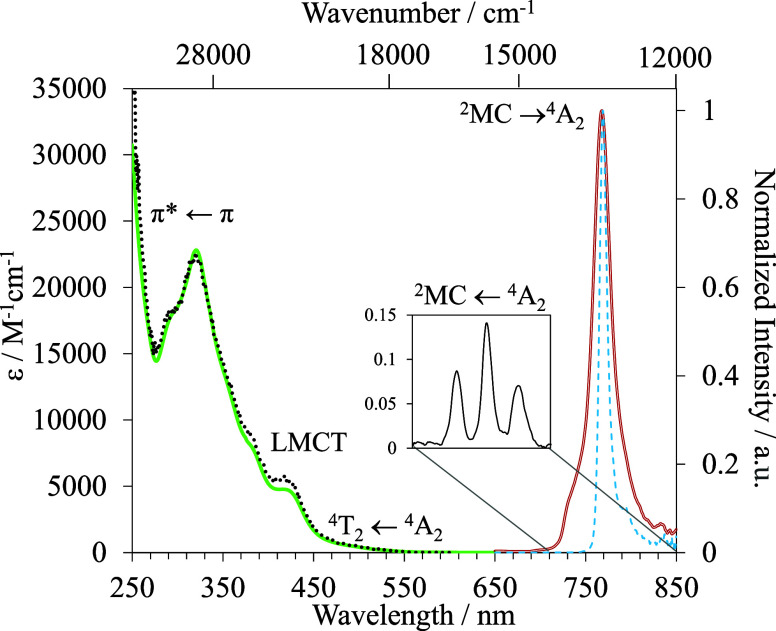
Absorption spectra (green trace), excitation spectrum (λ_em_ = 769 nm, dashed black trace), emission spectrum at room temperature (λ_exc_ = 400 nm, red trace), and emission spectrum at 77 K (λ_exc_ = 400 nm, dashed blue trace) of [Cr­(qpp)_2_]^3+^ in CH_3_CN at 3.3 × 10^–5^ M.

**Table 1:** Photophysical and Chiroptical Parameters

complex	λ_em_ ^a^ (nm) ^2^E(1) → ^4^A_2_	λ_em_ ^a^ (nm) ^2^T_1_(1) → ^4^A_2_	Δ_oct_ ^b^ (cm^–1^)	*B*^c^ (cm^–1^) *C*/*B* = 3.2/*C*/*B* = 4	β^d^	|*g*_lum_|^e^ ^2^T_1_ → ^4^A_2_	|*g*_lum_|^e^ ^2^E → ^4^A_2_
[Cr(dqp)_2_]^3+^	725	748	22,969	751:659	0.79	0.2	0.1
[Cr(dqp)(ddpd)]^3+^	729	764	23,353	732:642	0.77	0.14	0.07
[Cr(qpp)_2_]^3+^	729	769	23,283	728:639	0.76	0.11	0.08
[Cr(ddpd)_2_]^3+^	733	775	23,626	718:630	0.75	0.093	0.06

a λ_em_ from emission spectra at 293 K.

b Δ_oct_ extracted from CASSCF­(7,12)/FIC-NEVPT2 (Table S7).

c Racah parameter *B* assuming a fixed *C*/*B* ratio, *E*(*^4^T*_2_) = Δ_oct_, and *E*(*^2^T*_1_) = 9*B* + 3*C* – 24­(*B* ^2^/Δ_oct_).^29^ The *E*(^2^T_1_) state has been extracted from the emission spectrum at 77 K (Figure S5).

d Nephelauxetic parameter β (*B*/*B*_0_) where *B*_0_ is 950 cm^–1^ for free Cr^III^ ion in the gas phase.^17^

e Dissymmetry factor *g*_lum_ was determined at the emission band maximum.

Upon UV–vis excitation, HH-*rac*-[Cr­(qpp)_2_]^3+^ displays a sharp near-infrared (NIR) dual emission band with a maximum at 769 nm (13,003 cm^–1^) and a shoulder at 730 nm (13,698 cm^–1^). Similar dual SF emissions are observed within the complete family of chromium complexes with varying intensity ratios depending on the energy difference between these thermally equilibrated states (Figure S5). The excited state landscape was evaluated using the complete active space self-consistent field method (CASSCF­(7,12)/FIC-NEVPT2) for the four complexes. The calculations revealed a similar distribution of the excited states in the four compounds, where the microstates ^2^T_1_(1) and ^2^E­(1), derived from the lowering symmetry (*O* → *D*_2_), are the lowest energy excited states (Table S7 and Figure S18). Therefore, the dual emission originates from the Cr­(^2^T­(1) → ^4^A_2_) and Cr (^2^E­(1) → ^4^A_2_) transitions according to theoretical calculations, with the latter corresponding to the higher-energy one (Figures 3 and S18 and Table S7).

Finally, the associated excitation spectrum of HH-[Cr­(qpp)_2_]^3+^ closely matches its absorption spectrum (Figure 3), thus making this complex a good candidate for UV to NIR light-downshifting.

Beyond minor specific second-order corrections α*B* ^2^/Δ_oct_, the energy of both excited Cr­(^2^T_1_) and Cr­(^2^E) levels is mainly separated by 9*B* + 3*C* from the ground state Cr­(^4^A_2_) in pure octahedral complexes (*B* and *C* are the Racah parameters).^29^ Accordingly, the systematic red-shifts of the Cr­(^2^T­(1) → ^4^A_2_) and Cr­(^2^E­(1) → ^4^A_2_) transition observed along the series [Cr­(ddpd)_2_]^3+^ > [Cr­(qpp)_2_]^3+^ ≈ [Cr­(dqp)­(ddpd)]^3+^ > [Cr­(dqp)_2_]^3+^ (Table 1, columns 2 and 3 and Figure S5) suggest the operation of increasing nephelauxetic effect and larger electron delocalization which, as a first approximation, can be inferred from the decreasing value of the Racah parameter *B* (Table 1, column 5).^22,23^ It is important noticing that the Cr­(^2^T­(1) → ^4^A_2_) emission displays a more pronounced red-shift compared to the Cr­(^2^E­(1) → ^4^A_2_), indicating that the former transition is more influenced by changes in metal–ligand interactions (Figure S5). This difference has been attributed by Heinze and co-workers to the greater ability of the two paired electrons in the ^2^T­(1) state to delocalize through the ligand.^30,31^

Due to the weak distortion and strong ligand-field splitting, nonradiative deexcitation pathways are prevented and the Φ_PL_ reaches 8% in the absence of ^3^O_2_, which is in the range of the highest reported deuterium-free Cr^III^ molecular complexes (Table 2) with excited state lifetimes of τCr,obsE2′,T21′ = 1.41 ms at 77 K (Figure S6) and 422 μs at 298 K (Figures S7 and S8). Under air-equilibrated conditions, the Φ_PL_ drops to 0.4% and τCr,obsE2′,T21′ = 33 μs due to the presence of ^3^O_2_ in solution (Figures S9). The radiative rate for the low energy ^2^MC excited state, *k*_rad_, has been estimated assuming that the intersystem crossing between the ^4^MC and ^2^MC states is close to unity (i.e., *k*_rad_ = Φ/τ_obs_).^32^ The computed values for [Cr­(dqp)_2_]^3+^ (87 s^–1^) < [Cr­(dqp)­(ddpd)]^3+^ (93 s^–1^) < [Cr­(qpp)_2_]^3+^ (121 s^–1^) < [Cr­(ddpd)_2_]^3+^ (125 s^–1^) have been calculated in deaerated solutions for reliability (Table S12).^12,20^ Importantly, larger *k*_rad_ values are associated with less restricted spin-forbidden transitions and greater covalency.

**Table 2:** CPL Brightness (*B*_CPL_) Calculation for Each Emissive Transitions in Selected Di-tridentate Chromium Complexes

complex	ε/M^–1^ cm^–1^	ϕ_PL_/%^d^	^2^T_1_′/^2^E′ ratio		|*g*_lum_|	*B*_CPL_/M^–1^ cm^–1^
[Cr(dqp)_2_]^3+^	20,000​^a^	10.4	^2^E′	0.315	0.1	33
			^2^T_1_′	0.685	0.2	142
[Cr(ddpd)_2_]^3+^	30,000​^b^	12.1	^2^E′	0.136	0.06	13
			^2^T_1_′	0.864	0.093	146
[Cr(dqp)(ddpd)]^3+^	32,684​^c^	6.0	^2^E′	0.181	0.07	14
			^2^T_1_′	0.819	0.14	112
[Cr(qpp)_2_]^3+^	16,547​^c^	8.0	^2^E′	0.072	0.08	4
			^2^T_1_′	0.928	0.11	68

a λ_abs_ = 370 nm.

b λ_abs_ = 405 nm.

c λ_abs_ = 340 nm.

d Deaerated conditions.

### Chiral Resolution, Circular Dichroism, and Circularly Polarized Luminescence

Enantiomeric resolution of the racemic mixture HH-*rac*-[Cr­(qpp)_2_]^3+^ was achieved by using chiral stationary phase HPLC (CSP HPLC; Figure S10). Subsequent circular dichroism (CD) and circularly polarized luminescence (CPL) measurements were successfully carried out for both enantiomers (Figure 4a,b). Mirror images are consistently obtained in both CD and CPL experiments. The assignment to the *PP* and *MM* configurations was made possible by comparing their CD spectra with that of the analogue pure *MM*-[Cr­(dqp)_2_]^3+^ enantiomer, which could be crystallized.^13^ This assignment is further confirmed by theoretical calculations (Figure S20).

**Figure 4:**
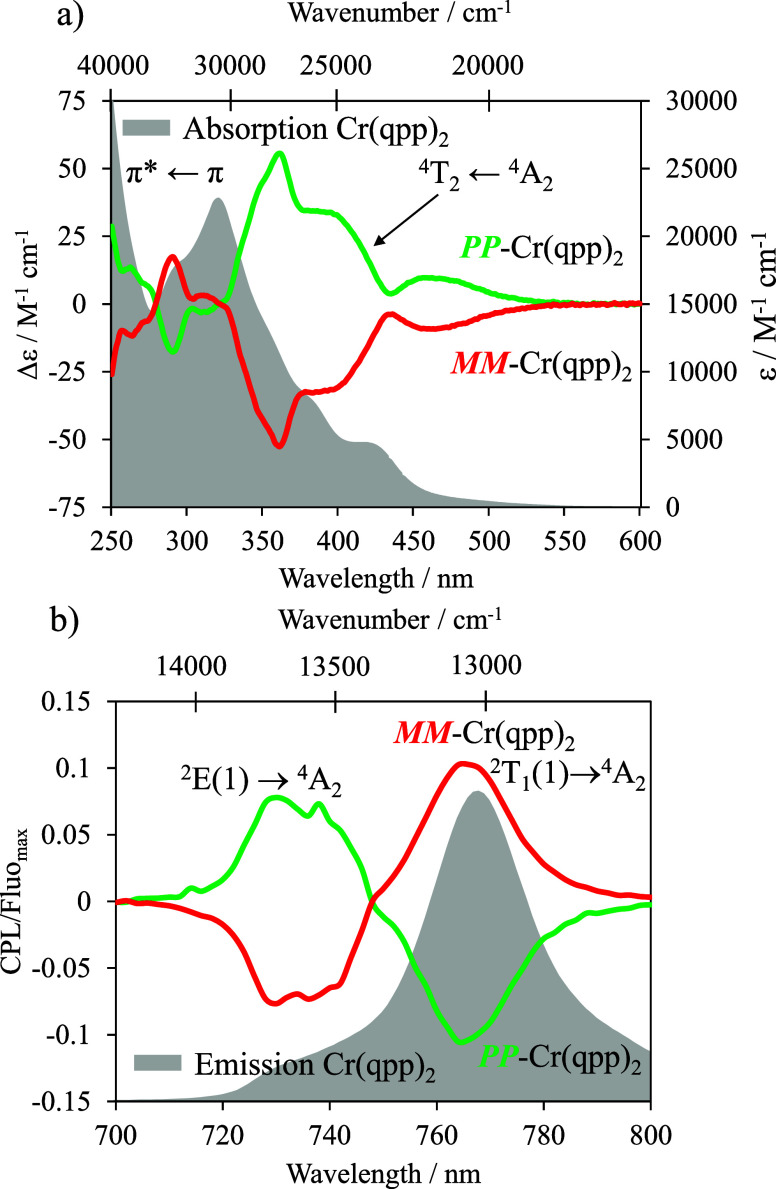
(a) CD spectra of both enantiomers HH-*PP*-[Cr­(qpp)_2_]^3+^ (green trace) and HH-*MM*-[Cr­(qpp)_2_]^3+^ (red trace) and corresponding absorption spectra (gray surface). (b) CPL spectra of both enantiomers HH-*PP*-[Cr­(qpp)_2_]^3+^ (green trace) and HH-*MM*-[Cr­(qpp)_2_]^3+^ (red trace) and corresponding emission spectra (gray surface). Recorded in EtOH/CH_2_Cl_2_ (1:1) at 293 K.

In the CD spectrum, a strong Cotton effect is observed within the 330–430 nm range reaching up to |Δε| = 55.6 M^–1^ cm^–1^ (Table S5), which can be attributed to the MC Cr­(^4^T_2_ ← ^4^A_2_) transition (Figure 4a). CPL measurements were recorded with an experimental bandwidth (EBW) of 2.4 nm to ensure a high resolution.^24^ Under unpolarized excitation (λ_exc_ = 350 nm), HH-*PP*/*MM*-[Cr­(qpp)_2_]^3+^ enantiomers display dual and strong circularly polarized emission in the NIR region (Figure 4b) with |*g*_lum_| = 0.11 for the Cr­(^2^T_1_(1) → ^4^A_2_) transition at 769 nm and 0.08 at 730 nm for Cr (^2^E­(1) → ^4^A_2_) (Figure S11). The CPL of the compounds *PP*/*MM*-[Cr­(dqp)_2_]^3+^ and *PP*/*MM*-[Cr­(dqp)­(ddpd)]^3+^ have been already measured by our group,^4,13^ whereas *PP*/*MM*-[Cr­(ddpd)_2_]^3+^ reported by Dee et al.^27^ has been re-evaluated in this work under identical experimental conditions to ensure consistency within the family of compounds. We found a dual CPL emission with |*g*_lum_| = 0.094 for the Cr­(^2^T_1_(1) → ^4^A_2_) and 0.06 for Cr­(^2^E­(1) → ^4^A_2_) transitions (Figure S12).

While they are of the same order of magnitude, subtle differences can be observed for the |*g*_lum_| values of the two transitions in all four chromium complexes (Table 1, columns 7 and 8). First, the isoelectronic [Cr­(dqp)­(ddpd)]^3+^ and [Cr­(qpp)_2_]^3+^ complexes display appreciable different CPL responses that might be related to different geometrical constraints resulting from the nature of the interstrand stacking interactions which are of homoleptic type in HH-[Cr­(qpp)_2_]^3+^ (quinoline–quinoline and pyridine–pyridine) and of heteroleptic type in [Cr­(dqp)­(ddpd)]^3+^ (twice quinoline–pyridine, Figures S2 and S3). Second, the estimated *g*_lum_ values for the Cr­(^2^T_1_(1) → ^4^A_2_) and Cr­(^2^E­(1) → ^4^A_2_) transitions within this family of complexes decrease as the SF emission undergoes a bathochromic shift (i.e., red-shift). This trend suggests a possible relationship between *g*_lum_ and the nephelauxetic effect which reflects metal-to-ligand electron delocalization.^33^ To explore this proposal, the Racah parameters *B* and *C*, taken as probes for the metal–ligand covalency and electronic delocalization, have been calculated assuming the relationships *C*/*B* = 3.2 and *C*/*B* = 4.^29,34,35^ (Table 1, column 5).^29,33^ Assuming a fixed *C*/*B* ratio simplifies the ligand-field analysis. However, detailed and systematic ligand-field studies of the electronic spectra of Cr^III^ compounds—and more recently Co^III^ complexes—have shown that *C*/*B* values can vary considerably, typically ranging from 3 to 10.^36,37^ Our primary focus of our work is on the correlation between the estimated *B* parameters and the experimental *g*_lum_ values. We believe that even though the absolute values of *B* may be affected by the chosen *C*/*B* ratio, the relative trends across the series are more robust, assuming that a consistent approach is applied throughout. The decrease of *B* along [Cr­(dqp)_2_]^3+^ (751 cm^–1^) > [Cr­(dqp)­(ddpd)]^3+^ (732 cm^–1^) ≈ [Cr­(qpp)_2_]^3+^ (728 cm^–1^) > [Cr­(ddpd)_2_]^3+^ (718 cm^–1^) implies some stepwise increase of metal–ligand covalency and electron delocalization upon replacement of terminal quinoline with *N*-methyl pyridine-2-amine units (Table 1), a trend confirmed by Ab Initio Ligand Field parameters computed from CASSCF­(3,5)/FIC-NEVPT2 (Table S6). The nephelauxetic effect is often quantified by the nephelauxetic parameter (β), which is the ratio between the Racah *B* parameter of a given ion in the gas phase (*B*_0_).^17^ For the reported compounds β ranges between 0.79 and 0.75 in going from [Cr­(dqp)_2_]^3+^to [Cr­(ddpd)_2_]^3+^ (Table 1 column 6). The concomitant decrease in *g*_lum_ (from 0.2 to 0.093) highlights the impact of the nephelauxetic effect on the chiroptical response of these complexes. The expanded delocalization of d-electrons due to a larger nephelauxetic effect (small *B* and β values) is expected to enhance mixing of the metal-ligand orbitals, thus partially relaxing the doubly forbidden character of the SF transition, further increasing the electric dipole transition moments (μ). Since *g*_lum_ is maximized when the electric dipole transition moment is minimized to become comparable with magnetic dipole transition moments (eq 1),^2^ its slight increase with nephelauxetic effect (covalency) should result in a decrease of the *g*_lum_ response (Table 1). To support this hypothesis, recent homoleptic pseudo-octahedral Cr^III^ complexes containing anionic tridentate 1,8-(bisoxazolyl)­carbazolide ligands and displaying very large nephelauxetic effect with Cr­(^2^T_1_,^2^E → ^4^A_2_) phosphorescence in the range of 813–845 nm have been shown to induce circularly polarized NIR emissions with unusually small *g*_lum_ in the scale of 2.0 × 10^–3^.^38^ Furthermore, Hasegawa and co-workers showed that the mixing of LMCT configurations into f–f excited states can perturb the electric and magnetic transition dipole moments, thereby affecting the CPL properties of chiral Eu^III^ complexes.^39,40^

Finally, *B*_CPL_, calculated as *B*_CPL_ = ε × ϕ_PL_ × 1/2 × |*g*_lum_|, is estimated for each transition (Table 2). Because *g*_lum_ is of opposite signs for Cr­(^2^T_1_(1) → ^4^A_2_) and Cr­(^2^E­(1) → ^4^A_2_) transitions (Figure 4b), a spectral deconvolution was necessary to accurately extract individual contributions to *B*_CPL_ (Figures S13–S15). This step is essential because the observed total emission (Figure S5) is a convolution of these two transitions, each contributing with different ϕ_PL_ and *g*_lum_. By resolving each transition’s spectral profile (^2^T_1_′/^2^E′ ratio in Table 2), we could accurately integrate the individual emission bands, determine their respective quantum yields, and subsequently calculate the corrected *B*_CPL_ values (Table 2). This deconvolution approach ensures a more reliable assessment of *B*_CPL_, avoiding misleading estimations due to spectral overlap and sign cancellation effects in *g*_lum_. Respectable values of *B*_CPL_ have been extracted for the new analogue [Cr­(qpp)_2_]^3+^ (Table 2, column 6) being some order of magnitude larger that most chiral organic molecules and chiral 4d and 5d-based metal complexes.^2^

## Conclusion

In conclusion, an up-to-now not recognized consequence of the nephelauxetic effect on the chiroptical properties of chromium­(III) complexes is suggested, with a particular focus on their *g*_lum_ and CPL behavior. Modulating metal–ligand covalency, particularly through ligand design, may influence the electronic structure and the magnitude of the transition dipole moments (μ and *m*). This plays a crucial role in the rational modulation of *g*_lum_ as it directly impacts the efficiency and alignment of chiroptical transitions. The red-shifted spin-forbidden emission controlled by the modulation of the nephelauxetic effect (i.e., the decrease of Racah *B* parameter) suggests that increased covalent character in the metal–ligand bond favors orbital mixing and decreases the *g*_lum_ factor. These findings offer new avenues for optimizing CPL properties in chromium­(III) complexes, distinguishing them from benchmark europium­(III) complexes by providing systems with improved luminescence efficiency without sacrificing the *g*_lum_ value.^41^ However, due to the limited number of examples in the literature, further studies involving a broader range of chromium­(III)-based complexes are necessary to fully explore the implications of this hypothesis.

## Supplementary Material



## Notes

Experimental details; theoretical calculations; list of transition bands; and decay curves (PDF)
