# Supplementary material for: Exploring the Role of the Nephelauxetic Effect in Circularly Polarized Luminescence of Chiral Chromium(III) Complexes
Source: J Am Chem Soc. 2025 Jun 28;147(27):23827–33. doi: 10.1021/jacs.5c06196 (PMC12257517; doi:10.1021/jacs.5c06196)
Supplement: Supplementary file 1 [file ja5c06196_si_001.pdf]

Exploring the Role of the Nephelauxetic Effect in Circularly Polarized Luminescence of Chiral Chromium(III) Complexes

Maxime Poncet, † Laura Cuevas-Contreras,‡ Yating Ye,‡ Laure Guénée,|| Carlos M. Cruz,+ Claude Piguet*† and Juan-Ramón Jiménez*‡

†Department of Inorganic and Analytical Chemistry, University of Geneva, quai E. Ansermet 30, CH-1211 Geneva 4, Switzerland.

‡ Departamento de Química inorgánica, Facultad de Ciencias, Unidad de Excelencia de Química Aplicada a Biomedicina y Medioambiente, Avda. Fuente Nueva s/n, 18071, Granada, Spain.

|| Laboratory of Crystallography, University of Geneva, quai E. Ansermet 24, CH-1211 Geneva 4, Switzerland

+Departamento de Química Orgánica, Facultad de Ciencias, Unidad de Excelencia de Química Aplicada a Biomedicina y Medioambiente, Avda. Fuente Nueva s/n, 18071, Granada, Spain.

Supporting Information

Table of contents

1. **EXPERIMENTAL SECTION** **5**

2. **SYNTHESIS** **6**

Synthesis of 6-bromo-N-methyl-N-(pyridin-2-yl)pyridin-2-amine 6

Synthesis of N-methyl-N-(pyridin-2-yl)-6-(quinolin-8-yl)pyridin-2-amine 7

Synthesis of $[Cr(qpp)_2](SO_3CF_3)_3$ 8

Scheme S1 Possible isomers and associated point groups for [Cr(N∩N∩N)2]3+ complexes where N∩N∩N is a tridentate symmetrical (ANA or BNB) or unsymmetrical (ANB) tridentate meridional chelate ligand). Bound planar tridentate ligands are referred to as ‘no helical twist’, whereas bound helically twisted ligands (possessing P or M helicity) are referred to as ‘helical twist’. 9

3. **X-RAY CRYSTALLOGRAPHY AND STRUCTURAL PROPERTIES** **10**

Table S1. Crystal data and structure refinement for $[Cr(qpp)_2](SO_3CF_3)_3{\cdot}0.5CH_3OH$.. 11

Figure S1. Ortep view of $[Cr(qpp)_2]^{2+}$ (thermal ellipsoids are drawn at 50% probability level) in the crystal structure of $[Cr(qpp)_2](SO_3CF_3)_3{\cdot}0.5CH_3OH$. Hydrogen atoms, counter ions and methanol molecules are omitted for clarity purpose. 12

Table S2. Selected bond lengths [Å] and angles [°] for $[Cr(qpp)_2](SO_3CF_3)_3{\cdot}0.5CH_3OH$. 13

Figure S2. (top) Interligand interplanar angle calculated for $[Cr(dqp)_2]^{3+}$, $[Cr(ddpd)_2]^{3+}$, $[Cr(dqp)(ddpd)]^{3+}$ and $[Cr(qpp)_2]^{3+}$. Angles are calculated in degrees (°). (down) Table summarizing the bite angle and distortion from perfect octahedron in selected di-tridentate chromium complexes 14

Figure S3. Difference in inter-ligand stacking in $[Cr(qpp)_2]^{3+}$ and $[Cr(dqp)(ddpd)]^{3+}$. For clarity, only the *PP* enantiomer of each complex is represented. 15

Table S3. SHAPE analysis of the compounds $[Cr(dqp)_2]^{3+}$, $[Cr(ddpd)_2]^{3+}$, $[Cr(dqp)(ddpd)]^{3+}$ and $[Cr(qpp)_2]^{3+}$ 15

4. **ELECTRONIC PROPERTIES** **16**

Table S4. Assignments and calculations from the absorption spectra (see Figure S4). Absorption spectra recorded at $3.3{\cdot}10^{-5}$ M in the UV-Vis region and at 3.3 mM between 650 and 850 nm at room temperature. Recording performed at 293 K in CH_3CN. 16

Figure S4. Deconvoluted spectra of the absorption of $[Cr(qpp)_2]^{3+}$ in CH_3CN at 3.3 mM 17

Figure S5. Emission spectra of the compounds $[Cr(dqp)_2]^{3+}$, $[Cr(ddpd)_2]^{3+}$, $[Cr(dqp)(ddpd)]^{3+}$ and $[Cr(qpp)_2]^{3+}$ a) at 293 K in acetonitrile and b) 77K in frozen acetonitrile. 18

5. **TIME RESOLVED EXPERIMENTS** **19**

Figure S6. Excited state lifetime fitting for the homoleptic complex $[Cr(qpp)_2]^{3+}$: $^2T_1(1)$ at 77 K in a H_2O/DMSO (1:1) solution at 10^{-4} M under air equilibrated conditions (detection at 767 nm, λ_{exc} = 355 nm Nd:YAG). 19

Figure S7. Excited state lifetime fitting for the homoleptic complex $[Cr(qpp)_2]^{3+}$: $^2T_1(1)$ at 298 K in a CH_3CN solution at 10^{-4} M under oxygen-free conditions (detection at 767 nm, λ_{exc} = 355 nm Nd:YAG). 19

Figure S8. Excited state lifetime fitting for the homoleptic complex $[Cr(qpp)_2]^{3+}$: $^2E(1)$ at 298 K in a CH_3CN solution at $3\cdot10^{-4}$ M under air equilibrated conditions (detection at 730 nm, λ_{exc} = 355 nm Nd:YAG). 20

Figure S9. Excited state lifetime fitting for the homoleptic complex $[Cr(qpp)_2]^{3+}$: $^2T(1)$ at 298 K in a CH_3CN solution at $3\cdot10^{-4}$ M under air equilibrated conditions (detection at 767 nm, λ_{exc} = 355 nm Nd:YAG). 20

6. CHIRAL RESOLUTION AND CHIROPTICAL PROPERTIES 21

Figure S10. CSP HPLC chromatograms of the enantiomeric resolution of HH-rac-$[Cr(qpp)_2]^{3+}$. A) Separation on the semi-preparative column (250 x 10 mm, 5 μm). B) and C) Reinjection of the collected fraction to confirm the enantiomeric excess of 100%ee on an analytical column (250 x 4.6 mm, 5 μm). 21

Table S5. Circular dichroism Cotton effect, the respective Δε and assignment in HH-$[Cr(qpp)_2]^{3+}$. 22

Figure S11. Circularly polarized luminescence spectra of the two enantiomers *PP*-$[Cr(qpp)_2]^{3+}$ (red) and *MM*-$[Cr(qpp)_2]^{3+}$ (green) in EtOH/DCM (1:1), displayed as dissymmetry factor g_{lum}. The grey area is the emission spectra of the racemic complex (λ_{exc} = 340 nm, experimental bandwidth = 2.4 nm). 22

Figure S13. (top) Deconvolution of the emission spectra of $[Cr(qpp)_2]^{3+}$ in two gaussian curves (red, green), the cumulative spectra fitting (blue) and the associated fitting results and parameters for the calculation of B_{CPL}. (down) Deconvolution of the emission spectra of $[Cr(ddpd)(dqp)]^{3+}$ in two gaussian curves (red, green), the cumulative spectra fitting (blue) and the associated fitting results and parameters for the calculation of B_{CPL}. 24

Figure S14. Deconvolution of the emission spectra of $[Cr(dqp)_2]^{3+}$ in two gaussian curves (red, green), the cumulative spectra fitting (blue) and the associated fitting results and parameters for the calculation of B_{CPL}. 25

Figure S15. Deconvolution of the emission spectra of $[Cr(ddpd)_2]^{3+}$ in two gaussian curves (red, green), the cumulative spectra fitting (blue) and the associated fitting results and parameters for the calculation of B_{CPL}. 25

7. THEORETICAL STUDIES 26

Figure S16. DFT optimized geometries of the quartet ground state of HH-*PP*-$[Cr(qpp)_2]^{3+}$. Spin density at the Cr center: 2.933361. 28

Figure S17. DFT optimized geometries of the quartet ground state of *PP*-$[Cr(dqp)(ddpd)]^{3+}$. Spin density at the Cr center: 2.931999. 28

Table S6. Ab Initio Ligand Field parameters computed from CASSCF(3,5)/FIC-NEVPT2 (in cm^{-1}). 29

Table S7. CASSCF(7,12)/FIC-NEVPT2 results. Energies in cm^{-1}. 29

Figure S18. Schematic representation of the energy levels for the calculated (CASSCF(7,12)/FIC-NEVPT2) excited states of related Cr^{III} complexes. 29

Table S8. Orbitals used in the CASSCF(7,12)/FIC-NEVPT2 calculations for HH-[Cr(qpp)$_2$]$^{3+}$. 30

Table S9. Orbitals used in the CASSCF(7,12)/FIC-NEVPT2 calculations for [Cr(dqp)(ddpd)$_2$]$^{3+}$........ 31

Table S10. Calculated 100 lowest electronic transitions for compound HH-[Cr(qpp)$_2$]$^{3+}$, their energies (in nm) and oscillator strength (in cgs units). Correction: –0.4 eV 32

Figure S19. Experimental UV-Vis spectrum of compound HH-*rac*-[Cr(qpp)$_2$]$^{3+}$ in CH_3CN and calculated oscillator strength of the calculated electronic transitions. 34

Figure S20. Experimental ECD spectrum of compound HH-*P,P*-[Cr(qpp)$_2$]$^{3+}$ in CH_3CN and calculated rotatory strength of the calculated electronic transitions. 34

Figure S21. TD-DFT charge transfer numbers of HH-[Cr(qpp)$_2$]$^{3+}$ defined from 0 to 1 of the first 100 electronic transitions. 35

Figure S22. Electron density difference maps (EDDMs) for the metal-centered transitions of HH-[Cr(qpp)$_2$]$^{3+}$. Blue: density loss; Purple: density gain. Isoval = 0.004 35

Table S11. Calculated 100 lowest electronic transitions for compound [Cr(dqp)(ddpd)]$^{3+}$, their energies (in nm) and oscillator strength (in cgs units). Correction: –0.4 eV 36

Figure S23. Experimental UV-Vis spectrum of compound *rac*-[Cr(dqp)(ddpd)]$^{3+}$ in CH_3CN and calculated oscillator strength of the calculated electronic transitions. 38

Figure S24. TD-DFT charge transfer numbers of [Cr(dqp)(ddpd)]$^{3+}$ defined from 0 to 1 of the first 100 electronic transitions. 39

Figure S25. Electron density difference maps (EDDMs) for the metal-centered transitions of [Cr(dqp)(ddpd)]$^{3+}$. Blue: density loss; Purple: density gain. 39

Table S12. Excited state lifetime, luminescence quantum yield and radiative rate (k_{rad}) values of the Cr(^{2}T(1)$\rightarrow$^{4}A$_2$) transition for the [Cr(dqp)$_2$]$^{3+}$, [Cr(ddpd)$_2$]$^{3+}$, [Cr(dqp)(ddpd)]$^{3+}$ and [Cr(qpp)$_2$]$^{3+}$complexes in aerated acetonitrile solution at 293 K. 40

1. EXPERIMENTAL SECTION

^{1}H and ^{13}C-NMR spectra were recorded at 298 K on a Bruker Avance 400 MHz spectrometer. Pneumatically-assisted electrospray (ESI) mass spectrum was recorded from 10^{-4} m solution on an Applied Biosystems API 150EX LC/MS System equipped with a Turbo Ionspray source®. Elemental analyses were performed by K. L. Buchwalder from the Microchemical Laboratory of the University of Geneva. Enantiopure materials were obtained by chiral stationary phase HPLC resolution on an Agilent 1260 Infinity II apparatus (quaternary pump, auto sampler, column thermostat and diode array detector) using a semi-preparative CHIRALPAK® IC column (250 x 10mm, 5µm) and HPLC grade solvents. Analytical runs after the separation were run in a analytical CHIRALPAK® IC / 250 x 4.6mm, 5µm. Absorption spectra in acetonitrile solution were recorded using a Lambda 1050 Perkin Elmer spectrometer (quartz cell path length 1 cm or 1 mm, 290-800 nm domain, 2×10^{-4} M and 650-800 nm domain, 7.7 mM). Emission spectra (excitation at 355 nm) and excitation spectra were recorded for either room temperature or frozen solution samples at 77 K in a Fluorolog (Horiba Jobin-Yvon), equipped with iHR320, a Xenon lamp 450 Watt Illuminator (FL-1039A/40A) and a water-cooled photo multiplier tube (PMT Hamamatsu R2658 or R928), and corrected for the spectral response of the system. For time-resolved experiments, the decay curves were recorded from previously excited samples at 77K and 293K, with a photomultiplier (Hamamatsu R2658 or R928) and a digital oscilloscope (Tektronix MDO4104C). Pulsed excitation at 355 nm was obtained with the third harmonic of a pulsed Nd:YAG laser (Quantel Qsmart 850). Low temperature (77 K) was achieved using liquid quartz transparent Dewar filled with liquid N_2 in the centre of which samples dissolved in acetonitrile were placed. Samples solutions were introduced into quartz tubes (4 mm interior diameter) and introduced in sample holder of the Dewar. The oxygen free decay curve measurements were recorded at 293 K from previously degassed acetonitrile solutions ($c \approx 10^{-4}$ M). Aerated solutions were prepared by using no-degassed acetonitrile as solvent. The emission quantum yield was measured according to an absolute method which makes use of an integrating sphere. Electronic circular dichroism (ECD) spectra were recorded on a Jasco J-815 spectropolarimeter at 20 °C in a 1 cm cuvette. The ECD spectra showed in this work are an average spectrum calculated after 3 scans (each one). For ECD measurements a fixed 0.1 s of integration time was selected. The Circularly Polarized Luminescence (CPL) were recorded with an Olis SOLO spectrophotometer. A fixed wavelength of 350 nm provided by a LED source was used, 1.0 s of integration time was selected, the CPL spectra showed in this work correspond to average spectra calculated after 10 scans.

2. SYNTHESIS

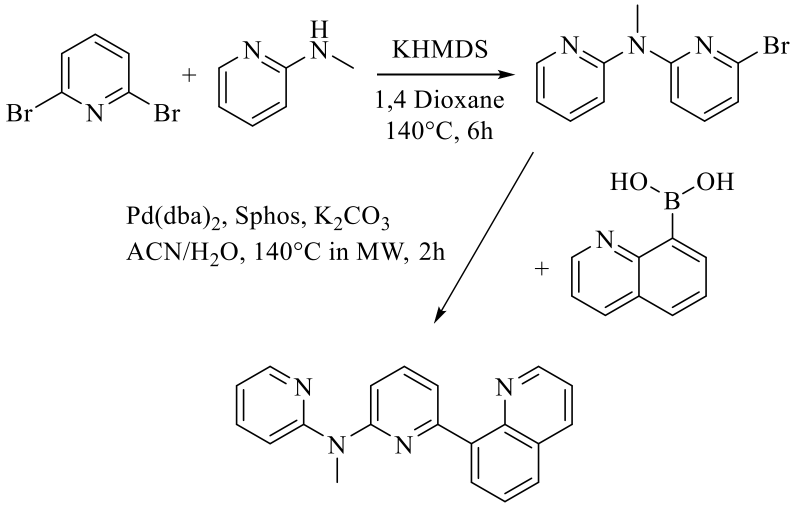

N-methyl-*N*-(pyridin-2-yl)-6-(quinolin-8-yl)pyridin-2-amine
qpp

Synthesis of 6-bromo-N-methyl-N-(pyridin-2-yl)pyridin-2-amine

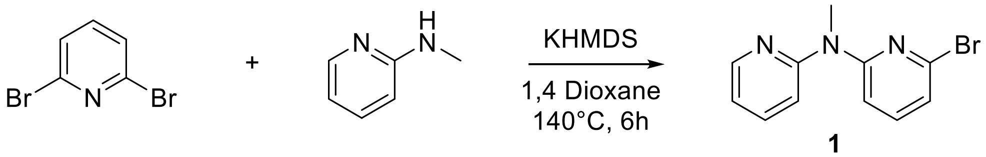

1

Potassium bis(trimethylsilyl)amide (1.02 g, 5.12 mmol, 1.72 equiv) was loaded in a flask and suspended in dioxane (5 mL) previously dried on molecular sieves. After the addition of *N*-methylpyridin-2-ylamine (0.348 mL, 2.97 mmol, 1.0 equiv), the solution turned yellow. 2,6-dibromopyridine (845.1 mg, 3.57 mmol, 1.20 equiv) was added. The mixture was heated at 140°C for 6h under MW radiations. After cooling to room temperature, water (250 mL) was added, and the aqueous phase was extracted three times with tetrahydrofuran/Et_2O (1:1, 3 x 100 mL). The combined organic phases were washed two times with a 1 M aqueous Na_2CO_3 solution and finally dried over $MgSO_4$. After removal of the solvent under reduced pressure, the product was purified by two consecutive column chromatography (silica gel, DCM 2% MeOH then second column cyclohexane/ethyl acetate 50:50). 6-bromo-N-methyl-N-(pyridin-2-yl)pyridin-2-amine **1** was isolated as a viscous liquid (419 mg, 45%). ^{1}H NMR (CD_2Cl_2, 400 MHz): δ 8.36 (dd, 1H), 7.63 (td, 1H), 7.47 (t, 1H), 7.34 (t, 1H), 7.24 (d, 1H), 6.71 (d, 2H), 7.08 (d, 1H), 6.97 (m, 1H), 3.56 (s, 3H). ESI-MS (CH_3CN) *m*/*z*: [**1**+H]$^+$ calc: 264.0, found: 264.3.

Synthesis of N-methyl-N-(pyridin-2-yl)-6-(quinolin-8-yl)pyridin-2-amine

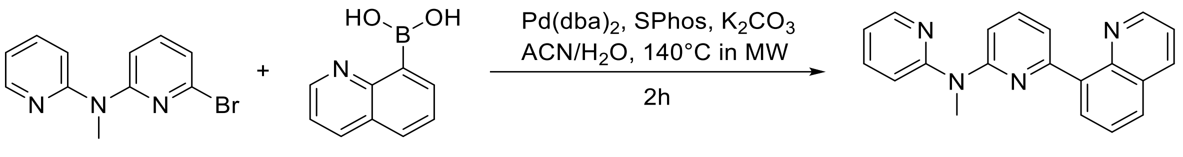

1 (411 mg, 1.56 mmol, 1.0 eq), Pd(dba)$_2$ (80 mg, 0.14 mmol, 0.09 eq), SPhos (112 mg, 0.27 mmol, 0.18 eq), K_2CO_3 (946 mg, 6.85 mmol, 4.38 eq) and quinoline-8-boronic acid (282 mg, 1.63 mmol, 1.04 eq) were weighed and put in a 20 mL MW vial. 10 mL ACN and 5 mL H_2O were added, and the solution was bubbled with argon for 15 min. The solution was put in the MW for 2h at 140°C. After the reaction, the vial was left to cool down at RT. Water and EtOAc were added, and the organic phase was extracted with water (3 x 250 mL). The organic fraction was dried over Na_2SO_4 and evaporated to dryness. Silica column chromatography cyclohexane/AcOEt 6:4 was done yielding N-methyl-N-(pyridin-2-yl)-6-(quinolin-8-yl)pyridin-2-amine qpp as a viscous translucid liquid (428 mg, 88%). ^{1}H NMR (CD_2Cl_2, 400 MHz): δ 8.95 (dd, 1H), 8.32 (d, 1H), 8.26 (dd, 1H), 8.16 (dd, 1H), 7.90 (dd, 1H), 7.80-7.63 (m, 3H), 7.54 (td, 1H), 7.46 (q, 1H), 7.33 (d, 1H), 7.22 (dd, 1H), 6.84 (ddd, 1H), 3.67 (s, 3H). ^{13}C(^{1}H) NMR (CD_2Cl_2, 400 MHz): δ 158.29 (s), 157.75 (s), 155.67 (s), 150.52 (s), 148.331 (s), 146.40 (s), 139.25 (s), 137.22 (s), 136.99 (s), 136.74 (s), 131.27 (s), 129.09 (s), 129.00 (s), 126.70 (s), 121.44 (s), 120.93 (s), 116.73 (s), 114.27 (s), 113.41 (s), 36.23 (s). ESI-MS (CH_3CN) *m/z*: [qpp+H]$^+$ calc: 313.3, found: 313.3.

Synthesis of [Cr(qpp)$_2$](SO$_3$CF$_3$)$_3$

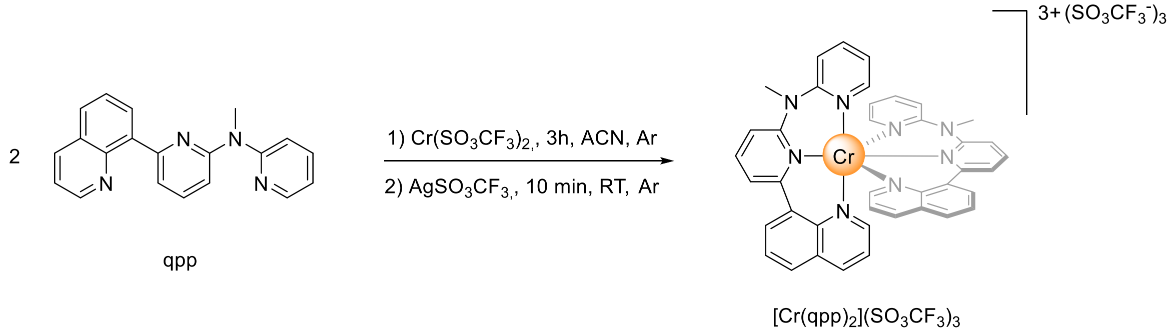

The ligand qpp was weighted outside of the glovebox in a 10 mL glass vial (108.4 mg, 0.35 mmol, 2 eq). Divalent chromium salt Cr(SO$_3$CF$_3$)$_2$ was weighted (66.9 mg, 0.17 mmol, 1 eq), dissolved in ACN (10 mL) and transferred onto the ligand. The reaction was left under stirring for 3h in the glovebox. Ag(I) triflate was then weighted (44.7 mg, 257 mmol, 1 eq) and added to the solution. Ag(0) is formed, precipitates, and the solution becomes orange due to the formation of Cr(III). The vial was taken out of the glovebox after 10 min. The solution was diluted with some ACN and then filtered to remove the Ag(0). The solution was evaporated to dryness. The solid was re-dissolved in the smallest amount of MeOH and Et$_2$O was slowly diffused yielding orange X-Ray quality crystals of the desired complex [Cr(qpp)$_2$](SO$_3$CF$_3$)$_3$ (Yield: 80%). ESI-MS (CH$_3$CN) *m*/*z*: [[Cr(qpp)$_2$](SO$_3$CF$_3$)$_2$]$^+$ calc: 974.1, found: 974.4; [[Cr(qpp)$_2$](SO$_3$CF$_3$)]$^{2+}$ calc: 412.6, found: 412.5; [Cr(qpp)$_2$]$^{3+}$ calc: 225.4, found: 225.4. Elemental analysis for **1**: $C_{43}H_{33}CrF_9N_8O_9S_3\cdot1.9H_2O$ %, found: C: 44.17, N: 9.24, H: 2.80; calculated: C: 44.56, N: 9.67, H: 3.2.

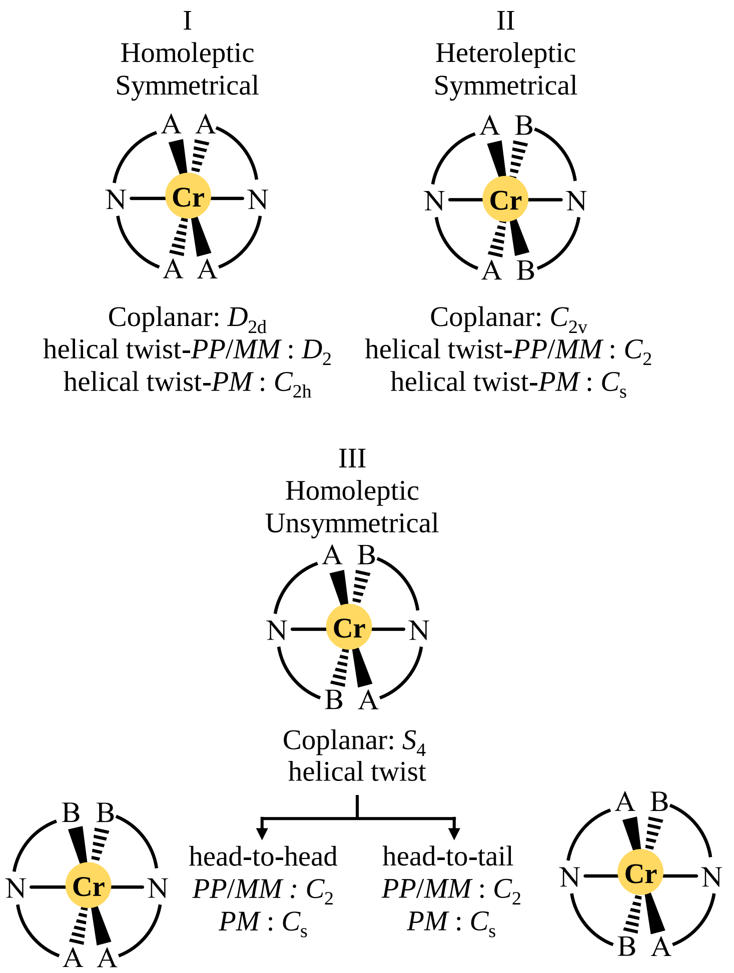

Scheme S1 Possible isomers and associated point groups for $[Cr(N\cap N\cap N)_2]^{3+}$ complexes where N∩N∩N is a tridentate symmetrical (ANA or BNB) or unsymmetrical (ANB) tridentate meridional chelate ligand). Bound planar tridentate ligands are referred to as ‘no helical twist’, whereas bound helically twisted ligands (possessing P or M helicity) are referred to as ‘helical twist’.

3. X-RAY CRYSTALLOGRAPHY AND STRUCTURAL PROPERTIES

Summary of crystal data, intensity measurements and structure refinements for $[Cr(qpp)_2](SO_3CF_3)_3 \cdot 0.5CH_3OH$ were collected in Table S1. Pertinent bond lengths, bond angles and interplanar angles were collected (Table S2 and Fig. S2) together with ORTEP view (Fig. S1). Crystals were mounted on Hampton cryoloops with protection oil. X-ray data collections were performed with a XtaLAB Synergy-S diffractometer equipped with a hybrid pixel array "hypix arc 150" detector. The structures were solved with ShelXT[S1] and all other calculations were performed with SHELXL,[S2] OLEX2[S3] and ORTEP[S4] programs. CCDC 2428511 contains the supplementary crystallographic data for this paper. These data can be obtained free of charge from The Cambridge Crystallographic Data Centre via www.ccdc.cam.ac.uk/data_request/cif.

References

S1 G. M. Sheldrick, *Acta Crystallogr. C: Structural Chemistry* **2015**, *71*, 3-8.

S2 G. M. Sheldrick, *Acta Crystallogr. A* **2008**, *64*, 112-122.

S3 O. V. Dolomanov, L. J. Bourhis, R. J. Gildea, J. A. K. Howard, H. Puschmann, *J. Appl. Crystallogr.* **2009**, *42*, 339-341.

S4 L. Farrugia, *J. Appl. Crystallogr.* **1997**, *30*, 565.

Table S1. Crystal data and structure refinement for $[Cr(qpp)_2](SO_3CF_3)_3{\cdot}0.5CH_3OH$.

CCDC number	CCDC 2428511	
Empirical formula	$C_{43.50}H_{34}CrF_9N_8O_{9.50}S_3$	
Chemical formula moiety	$C_{40}H_{32}CrN_8$, $3(CF_3O_3S)$, $0.5(CH_4O)$	
Formula weight	1139.96	
Temperature	120.00(10) K	
Wavelength	1.54184 Å	
Crystal system	Triclinic	
Space group	*P* -1	
Unit cell dimensions	*a* = 11.1427(2) Å	α= 89.6889(16)°.
	b = 12.5916(2) Å	β= 83.1154(17)°.
	c = 17.2523(4) Å	γ = 88.0330(15)°.
Volume	2401.69(8) $Å^3$	
Z	2	
Density (calculated)	1.576 Mg/m^3	
Absorption coefficient	4.072 mm^{-1}	
F(000)	1160	
Crystal size	0.19 x 0.08 x 0.04 mm^3	
Theta range for data collection	2.580 to 76.111°.	
Index ranges	$-12 \leq h \leq 13$, $-15 \leq k \leq 15$, $-21 \leq l \leq 21$	
Reflections collected	40063	
Independent reflections	9640 [R(int) = 0.0286]	
Completeness to theta = 67.684°	99.9 %	
Absorption correction	Analytical	
Max. and min. transmission	0.864 and 0.593	
Refinement method	Full-matrix least-squares on F^2	
Data / restraints / parameters	9640 / 1 / 748	
Goodness-of-fit on F^2	1.027	
Final R indices [I>2sigma(I)]	*R*1 = 0.0858, *wR*2 = 0.2435	
R indices (all data)	*R*1 = 0.0941, *wR*2 = 0.2505	
Extinction coefficient	n/a	
Largest diff. peak and hole	1.392 and -0.930 e.$Å^{-3}$	

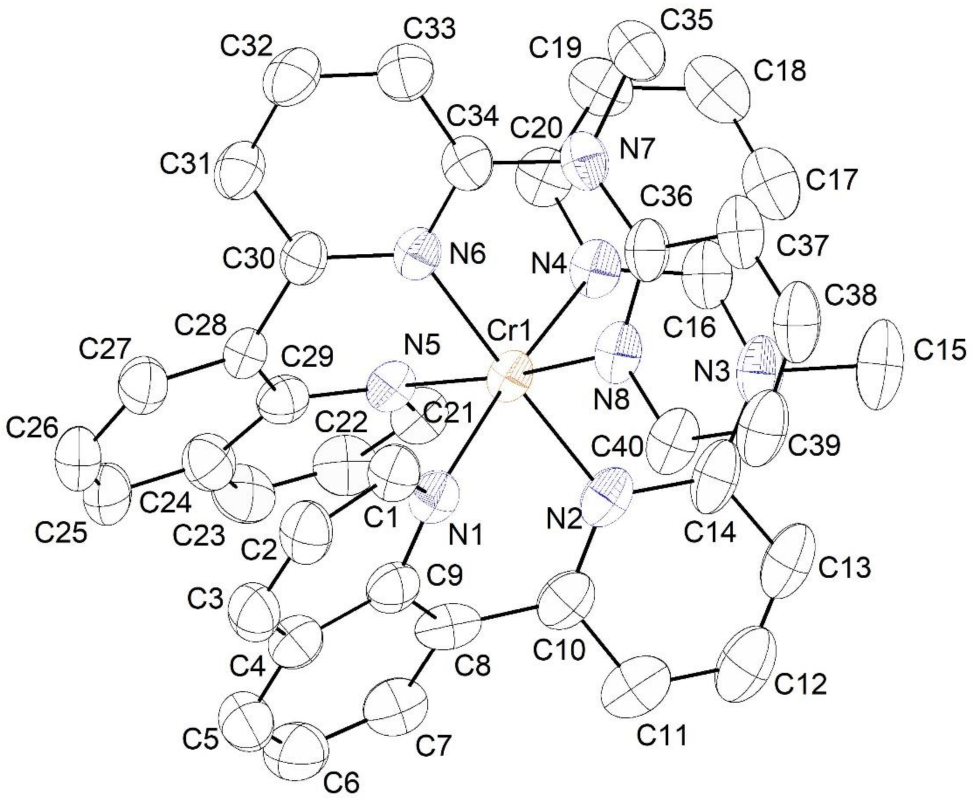

Figure S1. Ortep view of $[Cr(qpp)_2]^{3+}$ (thermal ellipsoids are drawn at 50% probability level) in the crystal structure of $[Cr(qpp)_2](SO_3CF_3)_3{\cdot}0.5CH_3OH$. Hydrogen atoms, counter ions and methanol molecules are omitted for clarity purpose.

<u>Comments on the crystal structure:</u>

<u>Disorder:</u>

Two triflates ions are disordered and modelled with two components (refined occupancies close to 0.5). Almost superimposed atoms O10-O11 and O12-F10 were refined with isotropic ADPs.

A methanol solvent molecule was located in the cell. Its occupancy factor was fixed to 0.5 to decrease ADPs down to reasonable values.

Table S2. Selected bond lengths [Å] and angles [°] for $[Cr(qpp)_2](SO_3CF_3)_3 \cdot 0.5CH_3OH$.

Cr(1)-N(1)	2.060(4)
Cr(1)-N(2)	2.038(4)
Cr(1)-N(4)	2.043(4)
Cr(1)-N(5)	2.064(4)
Cr(1)-N(6)	2.032(3)
Cr(1)-N(8)	2.041(4)
N(1)-Cr(1)-N(5)	92.19(15)
N(2)-Cr(1)-N(1)	88.38(16)
N(2)-Cr(1)-N(4)	85.88(16)
N(2)-Cr(1)-N(5)	93.63(15)
N(2)-Cr(1)-N(8)	92.61(15)
N(4)-Cr(1)-N(1)	174.27(15)
N(4)-Cr(1)-N(5)	88.21(15)
N(6)-Cr(1)-N(1)	92.37(14)
N(6)-Cr(1)-N(2)	178.71(16)
N(6)-Cr(1)-N(4)	93.37(15)
N(6)-Cr(1)-N(5)	87.38(14)
N(6)-Cr(1)-N(8)	86.37(15)
N(8)-Cr(1)-N(1)	88.10(15)
N(8)-Cr(1)-N(4)	92.12(15)
N(8)-Cr(1)-N(5)	173.76(14)

Symmetry transformations used to generate equivalent atoms:

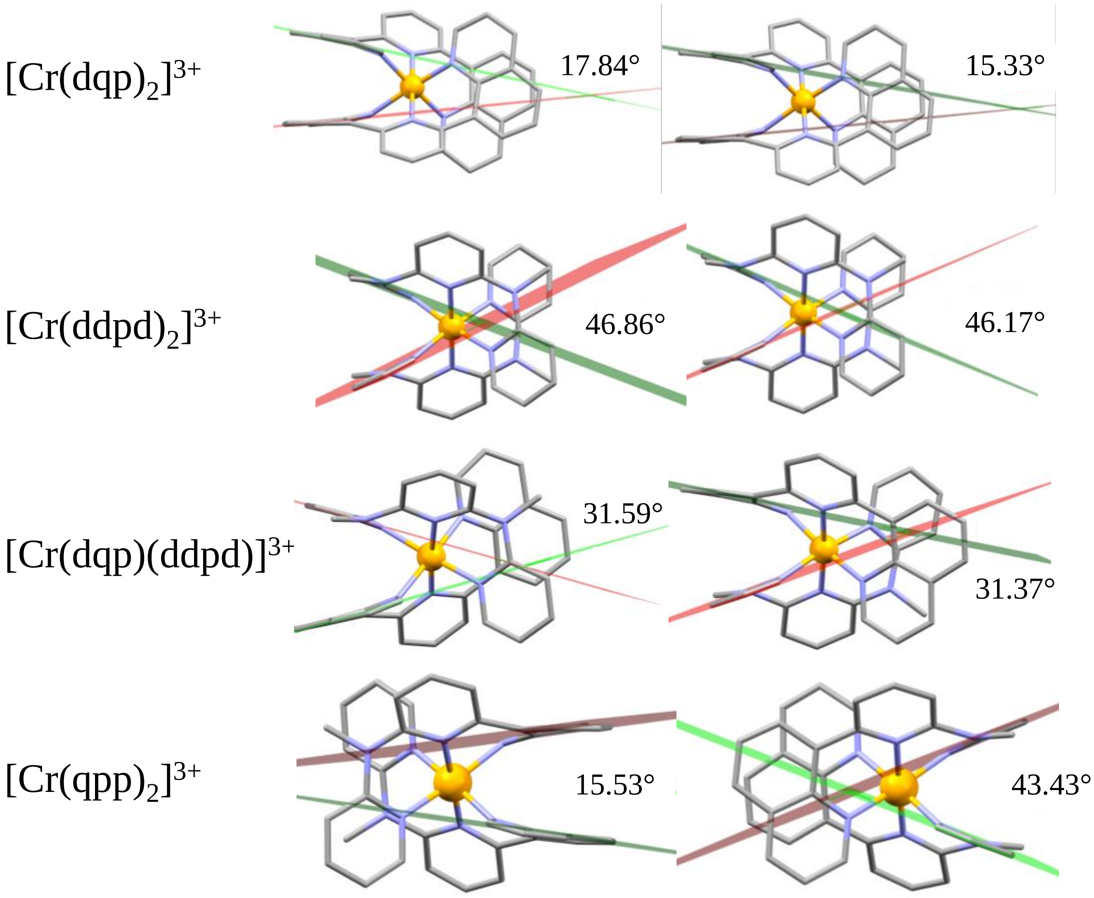

Complex	Cr-N average	Bite angle (°) [a]	Distortion from O_h (°) [b]	Interplanar angle (°) [c]		
				Quin-Quin	Quin-Pyr	Pyr-Pyr
$[Cr(dqp)_2]^{3+}$	2.060(5)	174.78(19)	28.94(10)	16.59(1.26)[d]	---	---
$[Cr(ddpd)_2]^{3+}$	2.044(5)	171.62(65)	37.11(53)	---	---	46.52(34)[d]
$[Cr(dqp)(ddpd)]^{3+}$	2.045(5)	174.35(113)	30.24(28)	---	31.48(11)[d]	---
$[Cr(qpp)_2]^{3+}$	2.047(3)	174.02(26)	31.97(24)	15.53[e]	---	43.43[e]

[a] Average value of the measurement of the bite angle N-Cr-N' within the same ligand [b] Distortion from perfect octahedron computed with $\Sigma = \sum_{i=1}^{12}|90-\varphi_i|$ in which φ_i is the cisoid bite angles N-Cr-N. [c] Average value of the interligand interplanar angles. [d] Average of the two angles. [e] Individual measurement.

Figure S2. (top) Interligand interplanar angle calculated for $[Cr(dqp)_2]^{3+}$, $[Cr(ddpd)_2]^{3+}$, $[Cr(dqp)(ddpd)]^{3+}$ and $[Cr(qpp)_2]^{3+}$. Angles are calculated in degrees (°). (down) Table summarizing the bite angle and distortion from perfect octahedron in selected di-tridentate chromium complexes

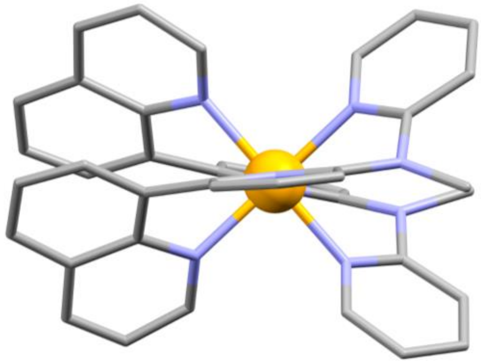

PP-$[Cr(qpp)_2]^{3+}$

Quinoline-Quinoline interaction
Pyridine-Pyridine interaction

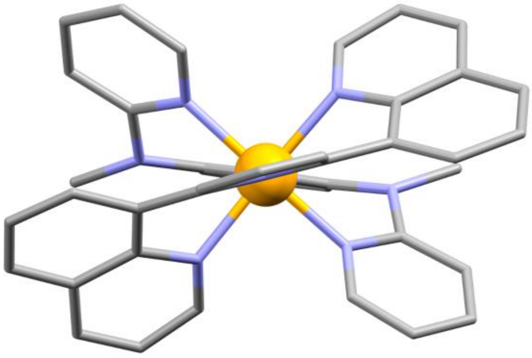

PP-$[Cr(dqp)(ddpd)]^{3+}$

2x Quinoline-Pyridine interaction

Figure S3. Difference in inter-ligand stacking in $[Cr(qpp)_2]^{3+}$ and $[Cr(dqp)(ddpd)]^{3+}$. For clarity, only the *PP* enantiomer of each complex is represented.

Table S3. SHAPE analysis of the compounds $[Cr(dqp)_2]^{3+}$, $[Cr(ddpd)_2]^{3+}$, $[Cr(dqp)(ddpd)]^{3+}$ and $[Cr(qpp)_2]^{3+}$

	OC-6 (Octahedron)
$[Cr(dqp)_2]^{3+}$	0.161
$[Cr(ddpd)_2]^{3+}$	0.335
$[Cr(dqp)(ddpd)]^{3+}$	0.250
$[Cr(qpp)_2]^{3+}$	0.213

4. ELECTRONIC PROPERTIES

Table S4. Assignments and calculations from the absorption spectra (see Figure S4). Absorption spectra recorded at $3.3 \cdot 10^{-5}$ M in the UV-Vis region and at 3.3 mM between 650 and 850 nm at room temperature. Recording performed at 293 K in CH_3CN.

Compound	λ (nm)	*v* (cm^{-1})	*ε* (M^{-1} cm^{-1})	Assignment [a]
$[Cr(qpp)_2]^{3+}$	292	34247	17757	π*← π
	322	31056	22716	π*← π
	354	28249	12942	π*← π
	384	26042	7716	π*← π
	425	23529	[b]	$^4T_2 \leftarrow {}^4A_2$
	698	14322	0.09	$^2T_1$’(3), ^2E’(2) $\leftarrow {}^4A_2$’
	731	13675	0.14	$^2T_1$’(2), ^2E’(1) $\leftarrow {}^4A_2$’
	765	13069	0.07	$^2T_1$’(1) $\leftarrow {}^4A_2$’

[a] Octahedral point group (O_h) is assumed for the $[Cr^{III}N_6]$ chromophore. [b] Not given because of the overlap with LMCT.

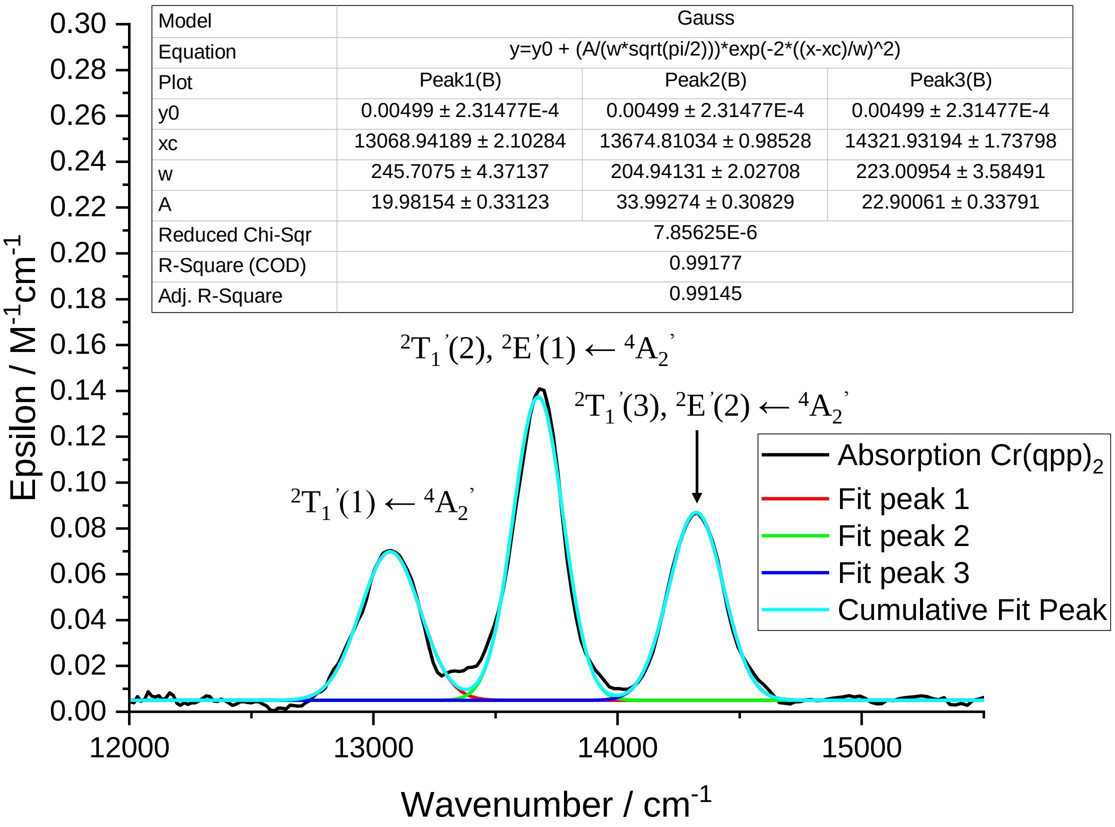

Figure S4. Deconvoluted spectra of the absorption of $[Cr(qpp)_2]^{3+}$ in CH_3CN at 3.3 mM

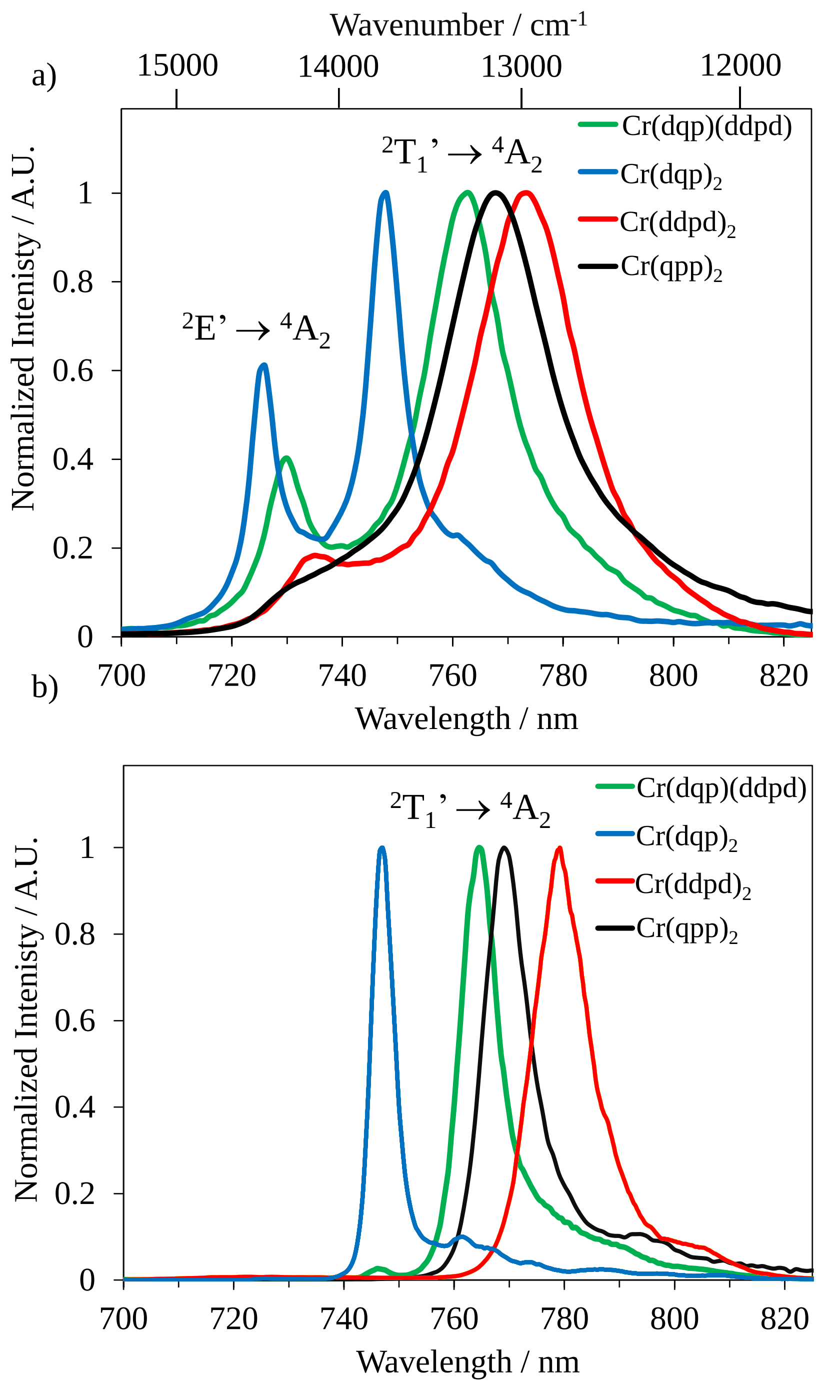

Figure S5. Emission spectra of the compounds $[Cr(dqp)_2]^{3+}$, $[Cr(ddpd)_2]^{3+}$, $[Cr(dqp)(ddpd)]^{3+}$ and $[Cr(qpp)_2]^{3+}$ a) at 293 K in acetonitrile and b) 77K in frozen acetonitrile.

5. TIME RESOLVED EXPERIMENTS

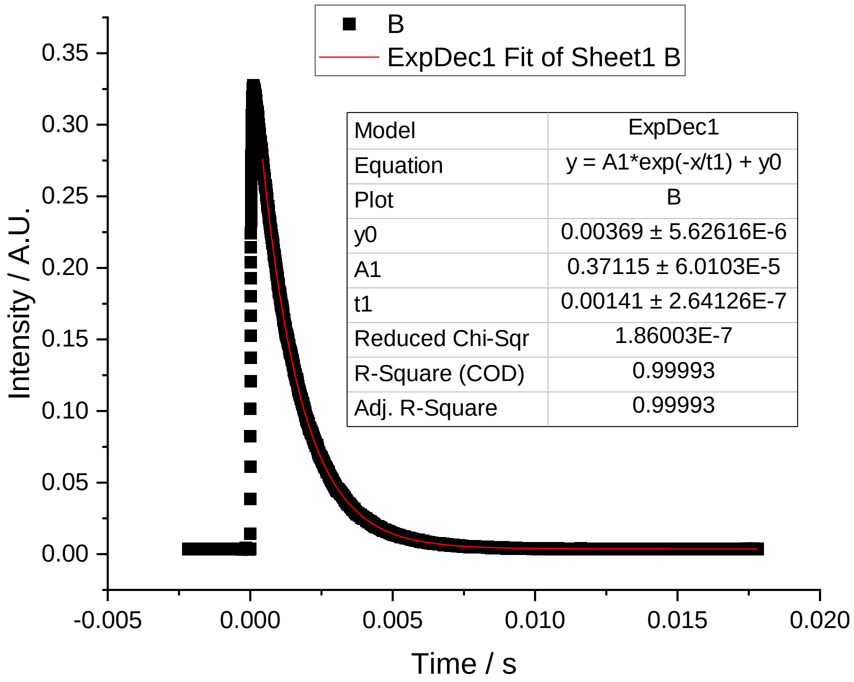

Figure S6. Excited state lifetime fitting for the homoleptic complex $[Cr(qpp)_2]^{3+}$: $^2T_1(1)$ at 77 K in a H_2O/DMSO (1:1) solution at 10^{-4} M under air equilibrated conditions (detection at 767 nm, λ_{exc} = 355 nm Nd:YAG).

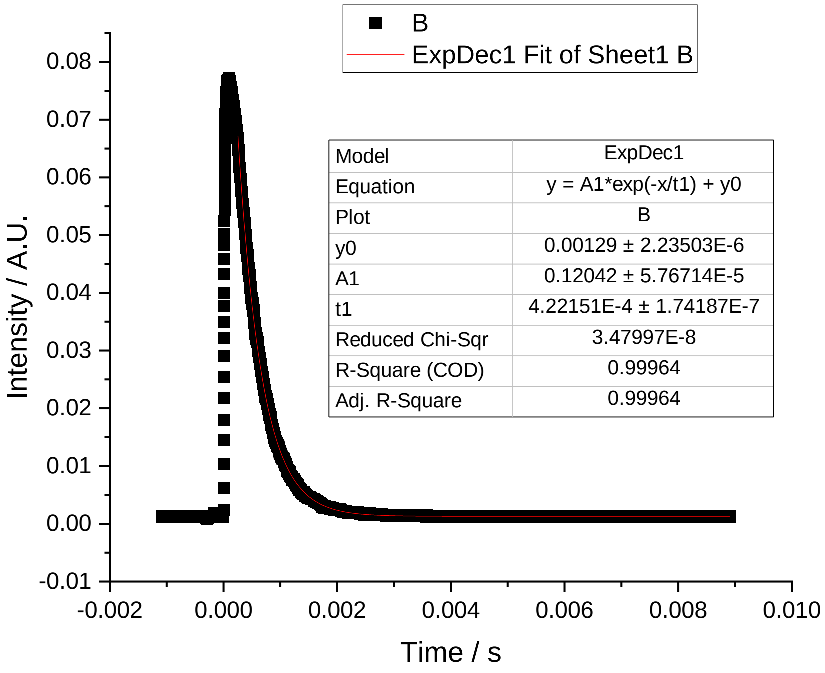

Figure S7. Excited state lifetime fitting for the homoleptic complex $[Cr(qpp)_2]^{3+}$: $^2T_1(1)$ at 298 K in a CH_3CN solution at 10^{-4} M under oxygen-free conditions (detection at 767 nm, λ_{exc} = 355 nm Nd:YAG).

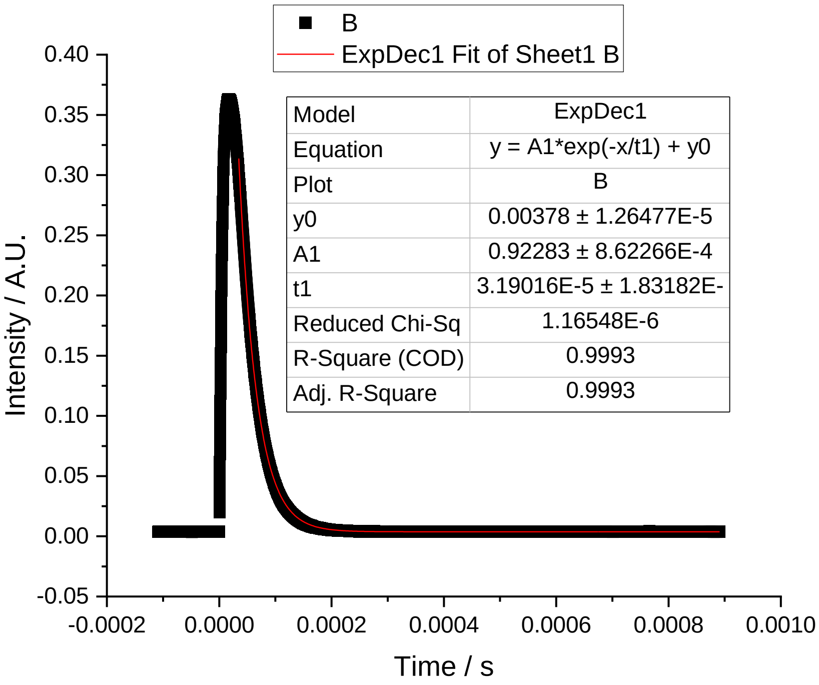

Figure S8. Excited state lifetime fitting for the homoleptic complex $[Cr(qpp)_2]^{3+}$: $^2E(1)$ at 298 K in a CH_3CN solution at $3\cdot10^{-4}$ M under air equilibrated conditions (detection at 730 nm, λ_{exc} = 355 nm Nd:YAG).

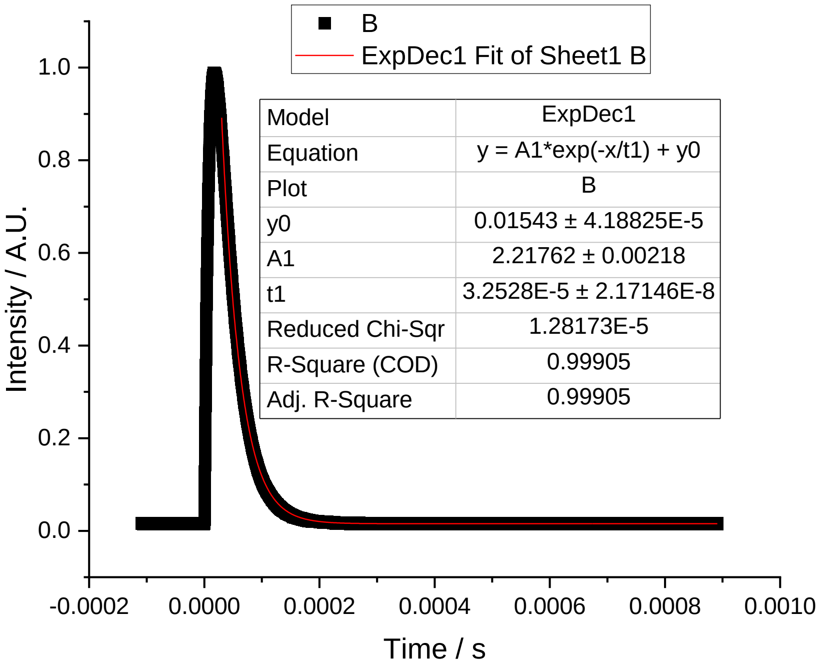

Figure S9. Excited state lifetime fitting for the homoleptic complex $[Cr(qpp)_2]^{3+}$: $^2T(1)$ at 298 K in a CH_3CN solution at $3\cdot10^{-4}$ M under air equilibrated conditions (detection at 767 nm, λ_{exc} = 355 nm Nd:YAG).

6. CHIRAL RESOLUTION AND CHIROPTICAL PROPERTIES

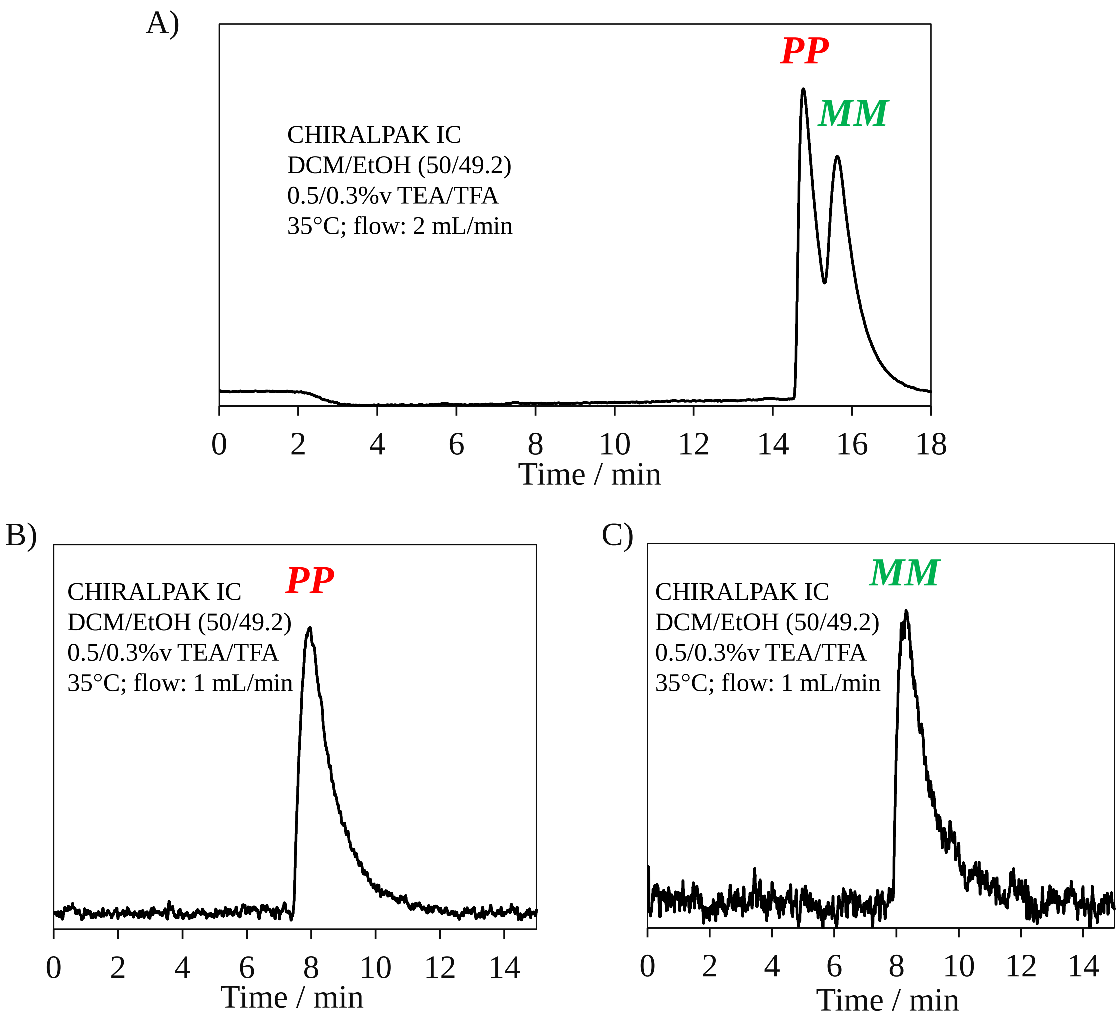

Figure S10. CSP HPLC chromatograms of the enantiomeric resolution of HH-rac-$[Cr(qpp)_2]^{3+}$. A) Separation on the semi-preparative column (250 x 10 mm, 5 μm). B) and C) Reinjection of the collected fraction to confirm the enantiomeric excess of 100%ee on an analytical column (250 x 4.6 mm, 5 μm).

Table S5. Circular dichroism Cotton effect, the respective Δε and assignment in HH-$[Cr(qpp)_2]^{3+}$.

Compound	λ (nm)	*v* (cm^{-1})	$\lvert\Delta\varepsilon\rvert$ (M^{-1} cm^{-1})	Assignment
	262	38168	9.4	$\pi^*\leftarrow\pi$
	290	34483	31.9	$\pi^*\leftarrow\pi$
	315	31746	55.6	$\pi^*\leftarrow\pi$
HH-$[Cr(qpp)_2]^{3+}$	348	28736	43.2	$\pi^*\leftarrow\pi$
	361	27701	2.2	LMCT
	401	24938	17.6	$^4T_2\leftarrow{}^4A_2$
	465	21505	13.6	LMCT

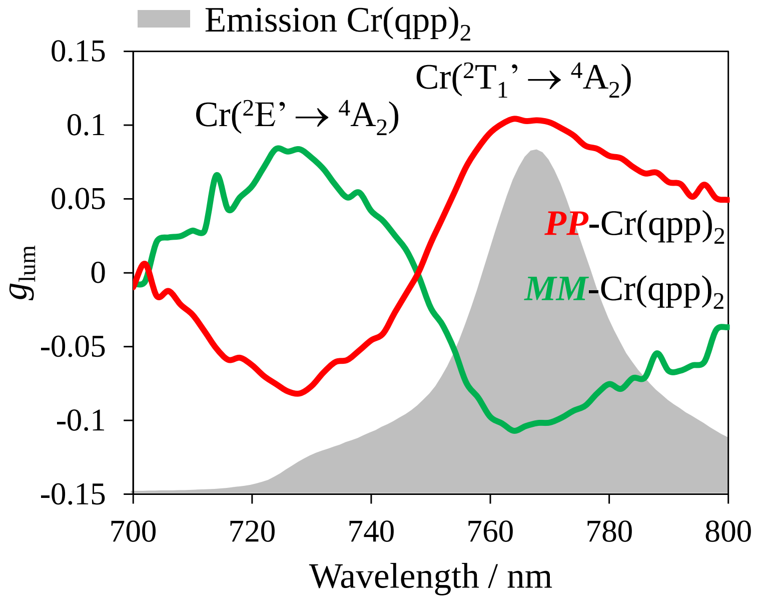

Figure S11. Circularly polarized luminescence spectra of the two enantiomers *PP*-$[Cr(qpp)_2]^{3+}$ (red) and *MM*-$[Cr(qpp)_2]^{3+}$ (green) in EtOH/DCM (1:1), displayed as dissymmetry factor g_{lum}. The grey area is the emission spectra of the racemic complex (λ_{exc} = 350 nm, experimental bandwidth = 2.4 nm).

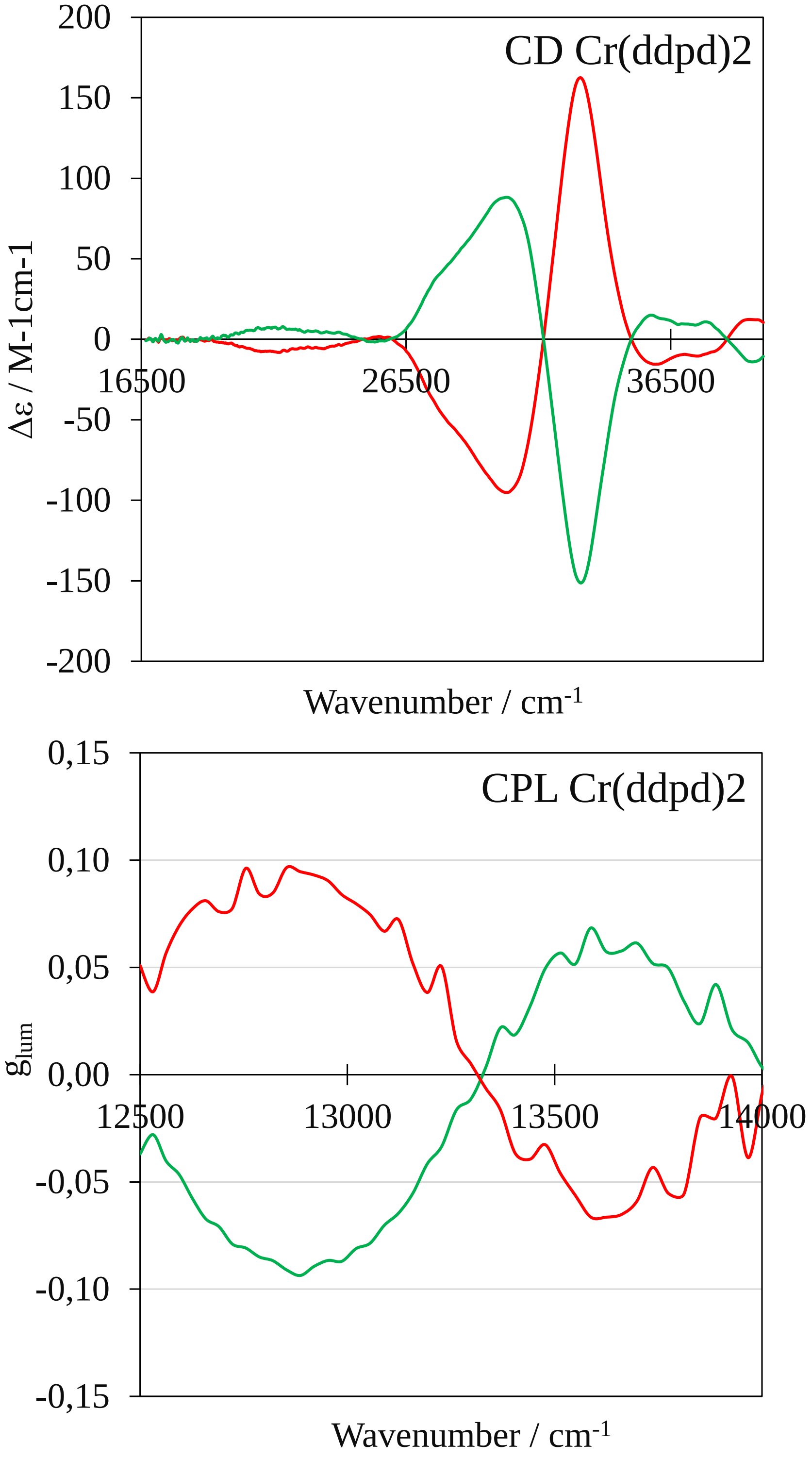

Figure S12. CD and CPL spectra of the two enantiomers PP-$[Cr(ddpd)_2]^{3+}$ and MM-$[Cr(ddpd)_2]^{3+}$ in EtOH/DCM (1:1), displayed as dissymmetry factor glum in the case of the CPL at λ_{exc} = 350 nm, experimental bandwidth = 2.4 nm.

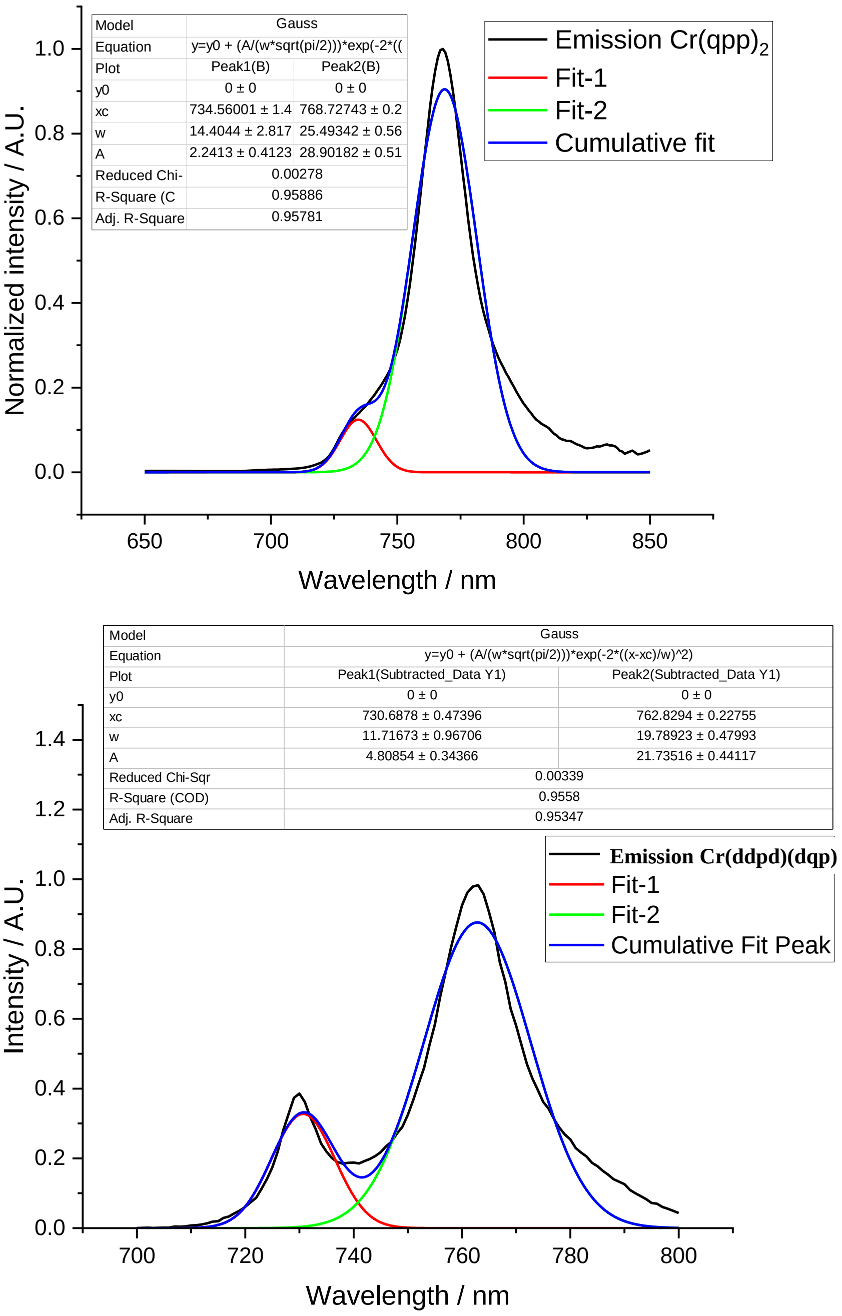

Figure S13. (top) Deconvolution of the emission spectra of $[Cr(qpp)_2]^{3+}$ in two gaussian curves (red, green), the cumulative spectra fitting (blue) and the associated fitting results and parameters for the calculation of B_{CPL}. (down) Deconvolution of the emission spectra of $[Cr(ddpd)(dqp)]^{3+}$ in two gaussian curves (red, green), the cumulative spectra fitting (blue) and the associated fitting results and parameters for the calculation of B_{CPL}.

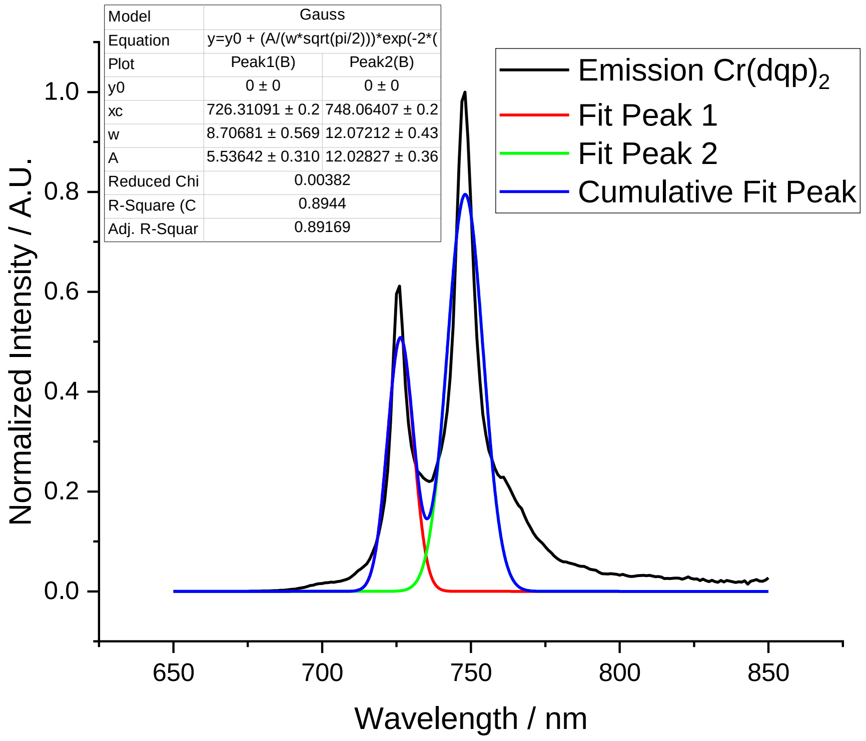

Figure S14. Deconvolution of the emission spectra of $[Cr(dqp)_2]^{3+}$ in two gaussian curves (red, green), the cumulative spectra fitting (blue) and the associated fitting results and parameters for the calculation of B_{CPL}.

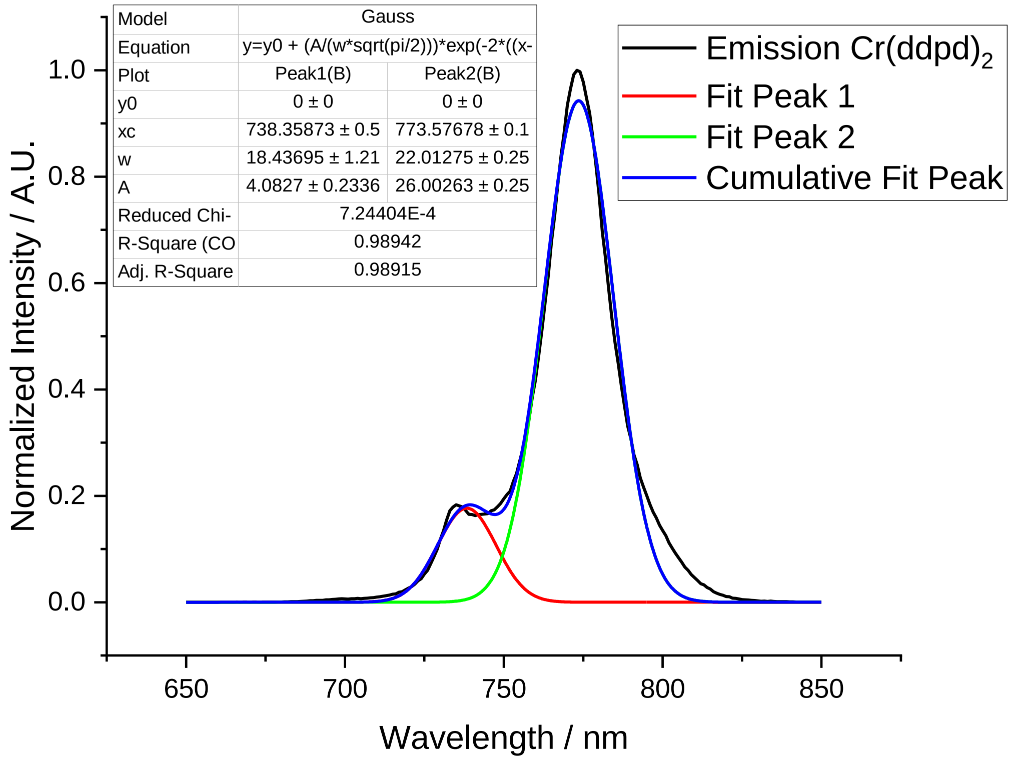

Figure S15. Deconvolution of the emission spectra of $[Cr(ddpd)_2]^{3+}$ in two gaussian curves (red, green), the cumulative spectra fitting (blue) and the associated fitting results and parameters for the calculation of B_{CPL}.

7. THEORETICAL STUDIES

The Orca[S5] (version 5.0.4) software package was used to investigate the structural and electronic properties of $[Cr(qpp)_2]^{3+}$, $[Cr(ddpd)_2]^{3+}$ and $[Cr(dqp)(ddpd)]^{3+}$ complexes. Starting from the X-ray diffraction structures, the ground, $^4A_2$ states for $[Cr(qpp)_2]^{3+}$, $[Cr(ddpd)_2]^{3+}$ and $[Cr(dqp)(ddpd)]^{3+}$ were obtained from DFT optimizations using the unrestricted version of the Becke three-parameters exchange function in combination with the Lee-Yang-Parr correlation functional (UB3LYP). The Ahlrichs' polarized valence triple-ζ basis set def2-TZVPP was used for these optimizations. The D3 version of Grimme's dispersion with Becke-Johnson damping (GD3BJ) was applied. Solvent effects were included via the Conductor-like Polarizable Continuum Model (CPCM) as implemented in Orca 5.0.4 with the dielectric constant of acetonitrile. Optimized geometries were confirmed to be stationary points by analysis of their vibrational frequencies. Tight convergence criteria were selected for the optimization step. The resolution of identity approach for the Coulomb term in combination with the chain-of-spheres approximation for the exchange term (RIJCOSX) was applied. The zero-order relativistic approximation (ZORA) was used to describe relativistic effects for $[Cr(qpp)_2]^{3+}$, $[Cr(ddpd)_2]^{3+}$ and $[Cr(dqp)(ddpd)]^{3+}$. Spin density information was extracted from the optimized geometries.

Ab initio ligand field (AILF) analysis was performed over the optimized geometries using Orca version 5.0.4. The complete-active-space self-consistent field method (CASSCF) together with the fully internally contracted N-electron valence perturbation theory to second order (FIC-NEVPT2) was used, selecting only the 3d orbitals as active space (CASSCF(3,5)/FIC-NEVPT2) and using the def2-TZVPP basis set in combination with the RI-JK approximation (def2/JK as auxiliary base). 10 quartet and 40 doublet roots were computed for the AILF analysis.

To accurately model the ligand field, CASSCF/FIC-NEVPT2 was used. Dominant bonding/antibonding orbitals formed between ligand and chromium and a second d shell were considered, creating an active space of 7 electrons and 12 orbitals (CASSCF(7,12)/FIC-NEVPT2). 10 quartet and 9 doublet roots were computed to calculate the energies of the excited states.

The 100 lowest energetic transitions were calculated by TD-DFT as implemented in Orca 5.0.4, using the unrestricted version of the CAM-B3LYP functional, selecting the def2-TZVPP basis set and considering solvent effects. Electronic transitions were corrected by –0.4 eV to better fit experimental results. Charge transfer numbers were calculated using TheoDore version 3.2.[S6]

References

[S5] a) F. Neese, *WIREs Comput. Mol. Sci.* **2012**, *2*, 73-78; b) F. Neese, *WIREs Comput. Mol. Sci.* **2022**, *12*, e1606.

[S6] F. Plasser, *J. Chem. Phys.* **2020**, *152*, 084108.

Optimized Geometries

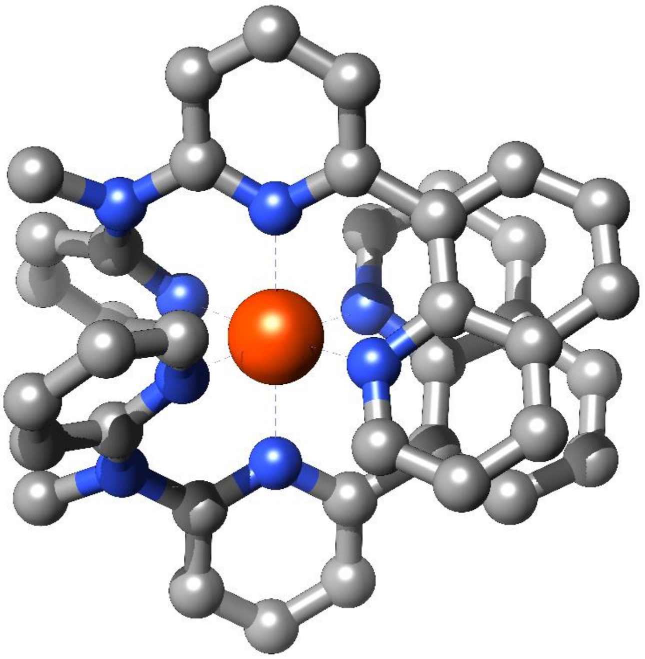

Figure S16. DFT optimized geometries of the quartet ground state of HH-*PP*-$[Cr(qpp)_2]^{3+}$. Spin density at the Cr center: 2.933361.

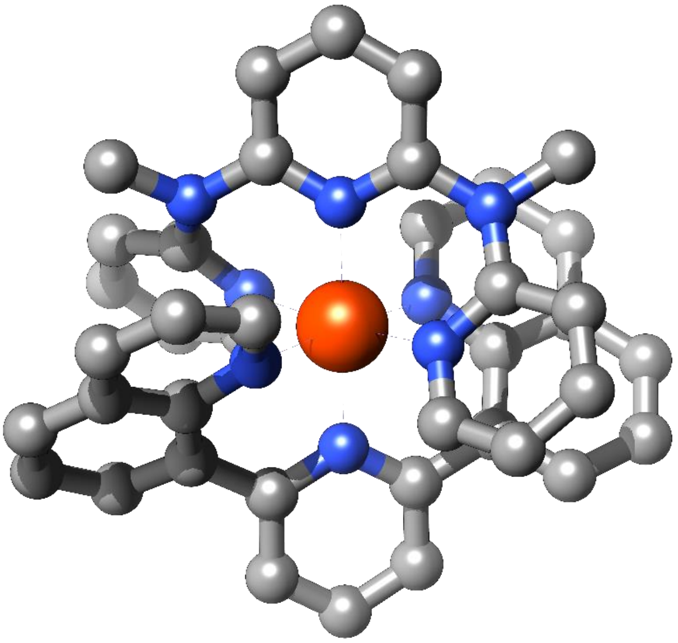

Figure S17. DFT optimized geometries of the quartet ground state of *PP*-$[Cr(dqp)(ddpd)]^{3+}$. Spin density at the Cr center: 2.931999.

Complete active space analysis of excited states

Table S6. Ab Initio Ligand Field parameters computed from CASSCF(3,5)/FIC-NEVPT2 (in cm^{-1}).

	B	*C*	*C/B*
$[Cr(qpp)_2]^{3+}$	978	2972	3.04
$[Cr(dqp)(ddpd)]^{3+}$	977	2979	3.05
$[Cr(ddpd)_2]^{3+}$ [a]	964	2972	3.08
$[Cr(dqp)_2]^{3+}$ [b]	982	2965	3.02

[a] Taken from: *J. Am. Chem. Soc.* **2021**, *143*, 11843-11855 (CASSCF(7,12)/FIC-NEVPT

[b] Taken from: *J. Mater. Chem. C* **2023**, *206*, 45-52.

Table S7. CASSCF(7,12)/FIC-NEVPT2 results. Energies in cm^{-1}.

	^2E (1)	^2E (2)	$^2T_1$ (1)	$^2T_1$ (2)	$^2T_1$ (3)	$^2T_2$ (1)	$^2T_2$ (2)	$^2T_2$ (3)	$^4T_2$ (1)	$^4T_2$ (2)	$^4T_2$ (3)
$[Cr(qpp)_2]^{3+}$	**15478**	15954	**15001**	15762	16000	23335	23869	24079	22584	23212	24055
$[Cr(dqp)(ddpd)]^{3+}$	**14980**	15467	**14629**	15920	16015	23332	23840	24034	22684	23102	24275
$[Cr(ddpd)_2]^{3+}$ [a]	**15355**	15916	**14769**	15647	15719	23328	23600	24033	22899	23230	24174
$[Cr(dqp)_2]^{3+}$ [b]	**15449**	16040	**15069**	15721	16002	23074	23992	24000	22051	23122	23736

[a] Taken from: *J. Am. Chem. Soc.* **2021**, *143*, 11843-11855 (CASSCF(7,12)/FIC-NEVPT)

[b] Taken from: *J. Mater. Chem. C* **2023**, *206*, 45-52.

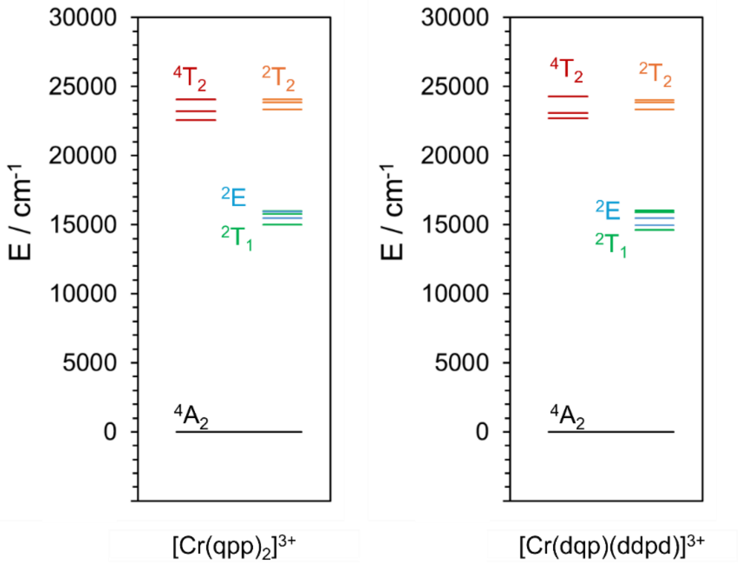

Figure S18. Schematic representation of the energy levels for the calculated (CASSCF(7,12)/FIC-NEVPT2) excited states of related Cr^{III} complexes.

Table S8. Orbitals used in the CASSCF(7,12)/FIC-NEVPT2 calculations for HH-[Cr(qpp)$_2$]$^{3+}$.

#	E (hartrees)	Orbital	#	Energy	Orbital
171	-0.861162	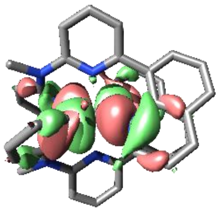	177	-0.181334	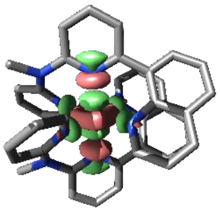
172	-0.862621	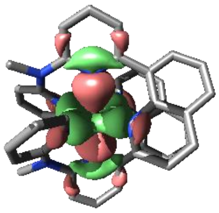	178	0.750359	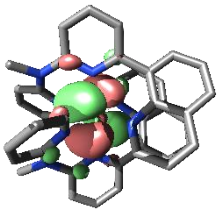
173	-0.408352	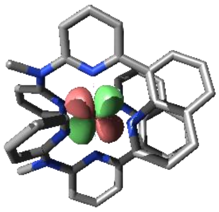	179	0.753764	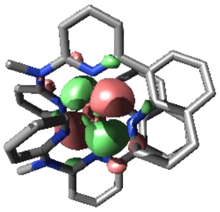
174	-0.396083	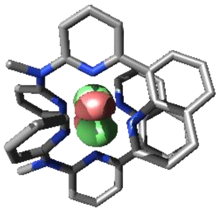	180	0.739318	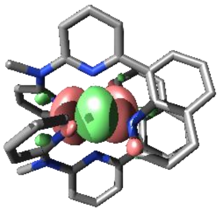
175	-0.396285	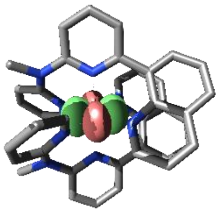	181	1.286427	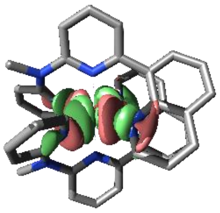
176	-0.193570	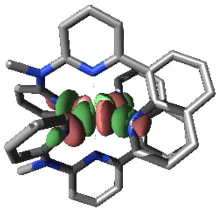	182	1.392694	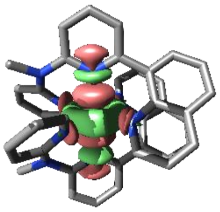

Table S9. Orbitals used in the CASSCF(7,12)/FIC-NEVPT2 calculations for $[Cr(dqp)(ddpd)_2]^{3+}$.

#	E (hartrees)	Orbital	#	Energy	Orbital
171	-0.862543	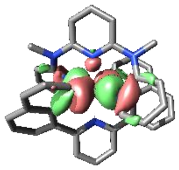	177	-0.179197	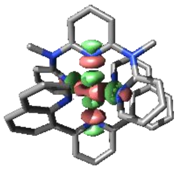
172	-0.860295	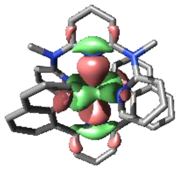	178	0.755608	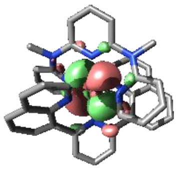
173	-0.407797	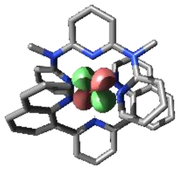	179	0.733884	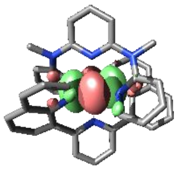
174	-0.395500	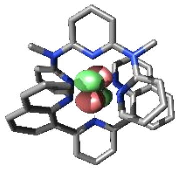	180	0.748743	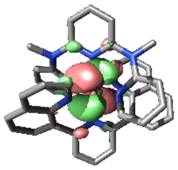
175	-0.395504	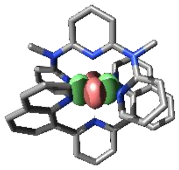	181	1.279793	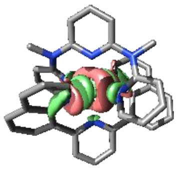
176	-0.193834	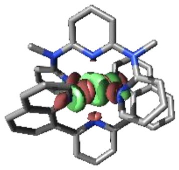	182	1.402372	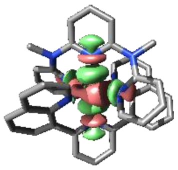

Electronic transitions

Table S10. Calculated 100 lowest electronic transitions for compound HH-$[Cr(qpp)_2]^{3+}$, their energies (in nm) and oscillator strength (in cgs units). Correction: –0.4 eV

#	λ / nm	*f* / cgs	*R* / 10^{40} cgs	#	λ / nm	*f* / cgs	*R* / 10^{40} cgs
1	514.0	0.000120241	2.34066	51	277.5	0.177481729	-168.23985
2	508.4	0.000878059	-4.45405	52	277.4	0.018700915	-18.99737
3	489.4	0.000084763	9.29553	53	272.0	0.003097457	15.28746
4	477.4	0.000024520	5.84886	54	271.3	0.000345698	0.76361
5	473.0	0.000287520	11.64818	55	268.7	0.050960484	-52.37483
6	410.2	0.019165025	-15.34178	56	268.3	0.009399547	5.83357
7	406.4	0.003771724	22.14982	57	265.6	0.028421288	0.21836
8	396.1	0.005162508	-13.50365	58	265.3	0.020208122	-3.01005
9	395.4	0.004625785	27.89708	59	264.8	0.000237603	-0.48217
10	394.9	0.000677421	0.92332	60	264.3	0.046358223	48.04911
11	390.1	0.002068283	-2.8722	61	262.3	0.010150547	8.24655
12	385.3	0.000346151	-4.43628	62	261.8	0.009632468	0.65115
13	384.9	0.000936640	-10.15542	63	261.7	0.004251306	8.59856
14	373.0	0.013569287	-6.2538	64	260.7	0.011215383	-27.38506
15	370.4	0.054145313	353.49215	65	260.0	0.019670412	61.75575
16	363.7	0.142562574	-98.50028	66	259.0	0.000182788	-3.17476
17	359.1	0.007381948	51.43868	67	258.1	0.022250318	35.35303
18	355.3	0.134857704	-158.44927	68	256.9	0.005430651	-6.22569
19	334.7	0.035682137	14.15074	69	256.0	0.054548339	86.36981
20	332.5	0.001586817	0.39325	70	254.7	0.004781361	-15.14595
21	331.4	0.157927063	-62.9708	71	254.4	0.003861045	-2.91343
22	328.8	0.033001968	314.21732	72	254.4	0.005348827	-15.24164
23	327.5	0.049635126	-15.66326	73	252.3	0.005355440	-17.63398
24	323.7	0.016725391	102.93319	74	251.1	0.002123504	9.64916
25	323.5	0.004318423	21.49515	75	249.8	0.016997107	41.37237
26	320.5	0.022902808	2.81885	76	249.6	0.047964305	-49.96965
27	319.3	0.000076958	-0.30491	77	249.1	0.009362685	-8.69371

28	318.3	0.027726653	35.33799	78	248.2	0.002580907	2.89404
29	317.0	0.035815519	-138.89015	79	246.1	0.008886370	36.26951
30	315.1	0.017701106	-11.15692	80	245.8	0.070785613	80.72715
31	312.6	0.157543298	-104.8175	81	243.9	0.005735586	36.70698
32	311.7	0.002191306	5.25832	82	243.5	0.018852627	-32.81495
33	307.1	0.039008423	-63.61002	83	243.2	0.000346060	0.6698
34	304.5	0.052174090	152.86269	84	243.2	0.003903986	-7.61035
35	303.2	0.066246954	-13.69296	85	241.9	0.016740802	-22.96547
36	302.4	0.031376385	14.25685	86	241.4	0.020741870	6.62137
37	297.6	0.001577823	17.65111	87	241.2	0.044763256	4.72862
38	297.2	0.002990051	-6.42455	88	239.8	0.002427718	14.18612
39	297.1	0.001829069	-2.32359	89	238.8	0.000760584	-7.16469
40	294.8	0.004923047	2.74897	90	238.7	0.072749393	196.73779
41	292.2	0.020139144	62.17626	91	238.3	0.007238295	-27.94327
42	291.2	0.099022948	-129.79126	92	237.4	0.053523100	24.70586
43	289.4	0.011656436	97.77763	93	237.3	0.038956522	-22.57289
44	287.9	0.000589719	-0.75407	94	236.2	0.060892468	13.87232
45	285.4	0.019510253	-6.64968	95	235.8	0.003135698	-5.15795
46	284.8	0.000159257	-1.84897	96	235.3	0.051468812	96.50238
47	283.2	0.009798317	-26.21437	97	235.1	0.010862157	32.47366
48	281.8	0.061859652	-75.99865	98	234.5	0.002380298	14.36999
49	280.6	0.018143318	1.9606	99	233.8	0.002312026	6.41934
50	278.4	0.000892939	-1.28954	100	232.8	0.036435283	45.75064

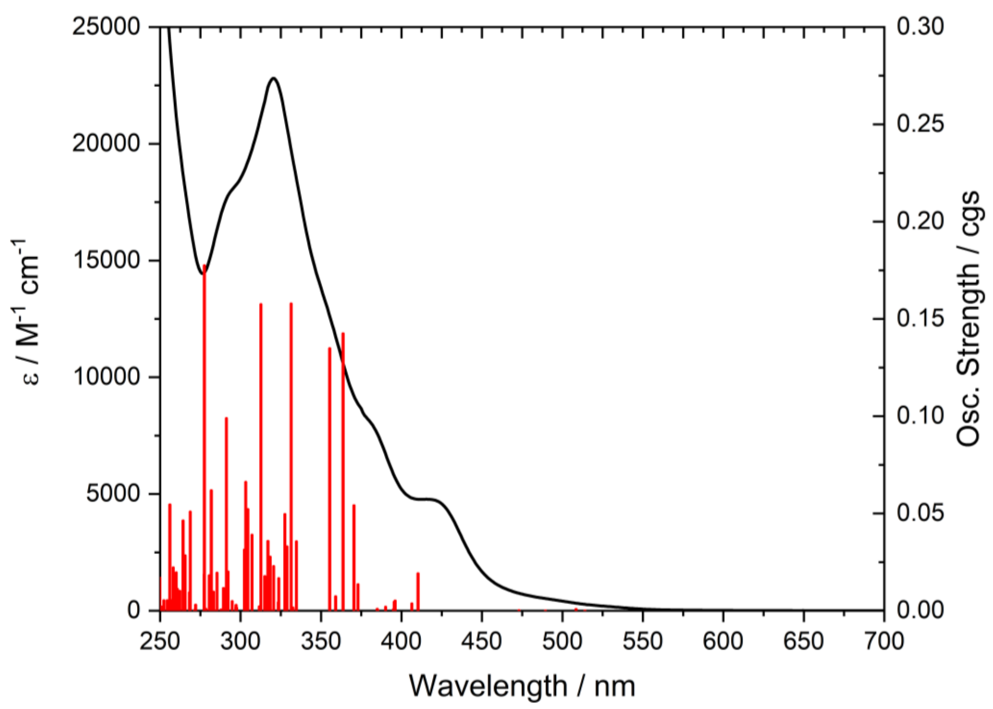

Figure S19. Experimental UV-Vis spectrum of compound HH-*rac*-$[Cr(qpp)_2]^{3+}$ in CH_3CN and calculated oscillator strength of the calculated electronic transitions.

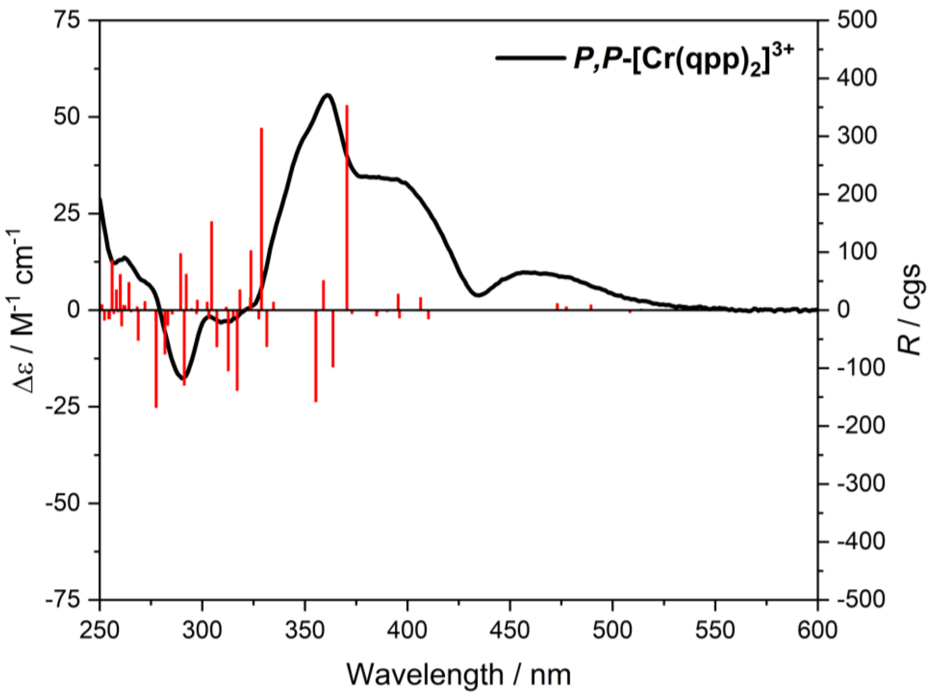

Figure S20. Experimental ECD spectrum of compound HH-*P,P*-$[Cr(qpp)_2]^{3+}$ in CH_3CN and calculated rotatory strength of the calculated electronic transitions.

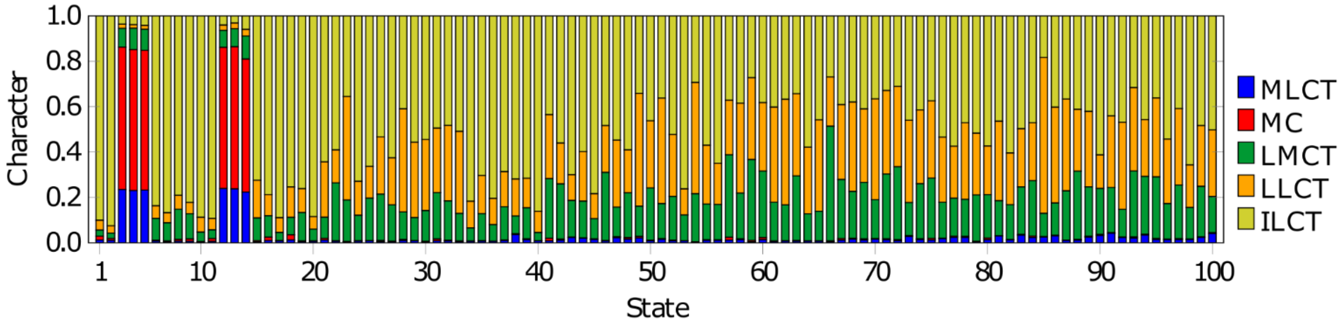

Figure S21. TD-DFT charge transfer numbers of HH-$[Cr(qpp)_2]^{3+}$ defined from 0 to 1 of the first 100 electronic transitions.

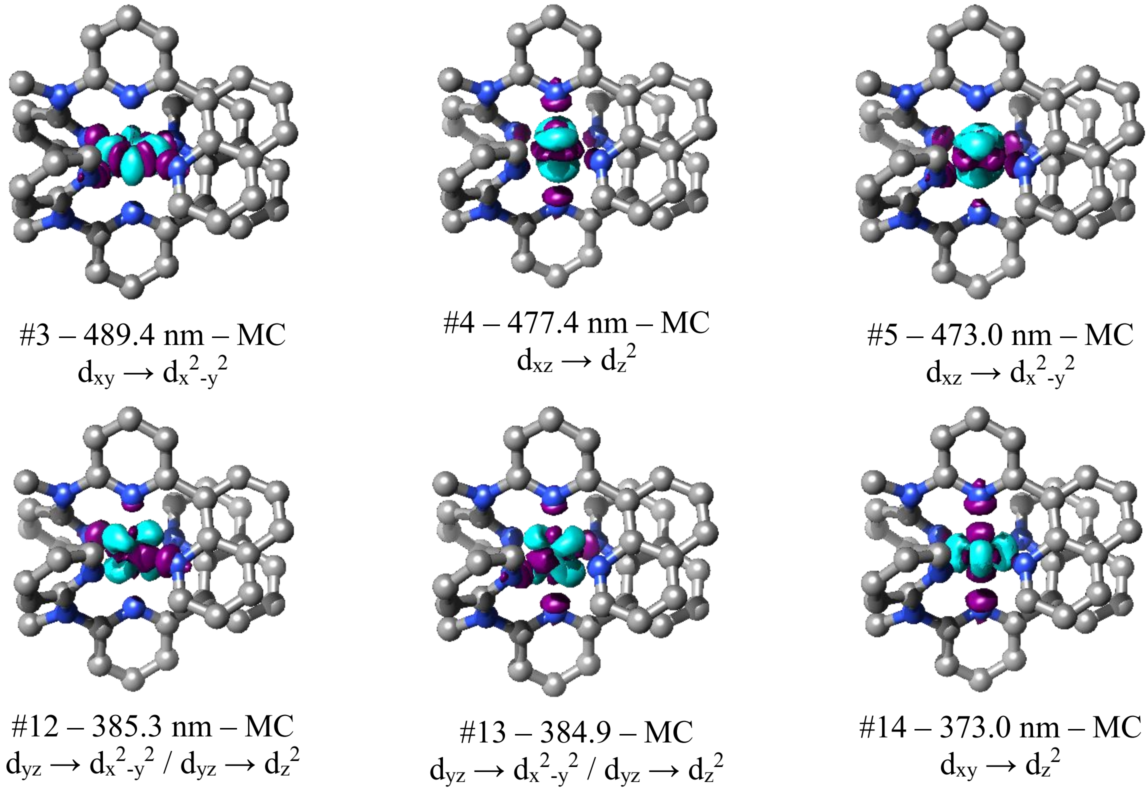

Figure S22. Electron density difference maps (EDDMs) for the metal-centered transitions of HH-$[Cr(qpp)_2]^{3+}$. Blue: density loss; Purple: density gain. Isoval = 0.004

Table S11. Calculated 100 lowest electronic transitions for compound $[Cr(dqp)(ddpd)]^{3+}$, their energies (in nm) and oscillator strength (in cgs units). Correction: –0.4 eV

#	λ / nm	*f* / cgs	*R* / 10^{40} cgs	#	λ / nm	*f* / cgs	*R* / 10^{40} cgs
1	514.5	0.000781476	-3.51447	51	274.8	0.001881370	0.48368
2	507.0	0.000003322	0.18364	52	274.4	0.001118102	4.32436
3	486.9	0.000038541	6.9026	53	273.1	0.046759987	51.22923
4	483.5	0.000619485	23.77243	54	271.5	0.024780057	-5.28952
5	463.5	0.000427797	2.62753	55	270.4	0.130536334	-128.69843
6	412.2	0.011916339	-4.5966	56	267.1	0.048442105	-27.04037
7	403.1	0.005446011	-9.21515	57	266.0	0.018751325	-2.27902
8	394.3	0.000641727	9.29716	58	265.8	0.001017854	-4.16279
9	391.9	0.006786639	4.20604	59	263.6	0.027689251	25.93941
10	391.6	0.000392232	-0.13058	60	260.8	0.000733672	0.56127
11	389.1	0.001101865	1.17282	61	259.9	0.036926641	-40.39076
12	385.0	0.032594504	3.90702	62	259.3	0.006384768	31.11278
13	377.7	0.003109589	-11.75627	63	257.6	0.007839077	-29.42939
14	372.4	0.000108211	0.92544	64	256.9	0.000824197	-8.646
15	368.1	0.000111871	-0.16708	65	254.9	0.015677384	41.43054
16	359.1	0.003175449	-7.48287	66	253.7	0.005551679	6.2579
17	358.1	0.543515840	32.29098	67	253.3	0.042290536	-15.2823
18	348.8	0.030233273	226.43445	68	253.0	0.003683614	-1.3936
19	342.8	0.019682327	-18.99448	69	252.5	0.010497049	18.59619
20	337.9	0.000618653	8.47326	70	250.5	0.014285976	11.85614
21	334.8	0.000062242	0.36261	71	250.4	0.026834153	13.74466
22	331.2	0.000374557	0.76756	72	250.2	0.008270560	17.43628
23	327.7	0.016944435	11.87063	73	249.5	0.003855234	4.46921
24	327.4	0.013737318	9.84195	74	248.7	0.004645958	9.00089
25	325.3	0.053486830	120.34529	75	248.4	0.038984317	-26.05312
26	319.0	0.017216217	-2.07659	76	248.2	0.034892142	20.14469
27	317.3	0.019073900	-22.4918	77	246.8	0.016322536	24.54944
28	315.3	0.001852237	-3.31956	78	245.9	0.003395026	12.82024
29	313.1	0.025905940	-48.73881	79	245.4	0.017717517	-16.23705

30	309.5	0.036909863	-66.35018	80	244.5	0.020039173	61.99754
31	308.6	0.004714937	41.81705	81	243.7	0.000951659	-7.99384
32	307.1	0.103025277	356.324	82	243.3	0.003481376	-6.43819
33	306.1	0.000281468	0.80077	83	242.3	0.015740667	-7.31644
34	304.5	0.009537171	-44.95987	84	241.2	0.004344722	15.06776
35	303.7	0.186627404	-267.36988	85	240.9	0.009984850	45.54568
36	300.4	0.007042920	-0.64618	86	240.5	0.015933692	-10.2635
37	299.5	0.010970981	-3.62861	87	239.6	0.000646995	-1.48515
38	296.2	0.001437071	0.07126	88	239.4	0.045282398	-78.34039
39	295.5	0.170108637	46.58715	89	238.9	0.003604560	-13.88936
40	294.0	0.000493529	-1.42583	90	237.6	0.020235528	-8.33077
41	289.5	0.003271206	2.21842	91	237.3	0.009237615	19.76688
42	288.2	0.007537634	20.22585	92	236.6	0.025694306	-10.19035
43	286.7	0.000016202	0.22945	93	235.8	0.000296598	-1.3133
44	284.2	0.001541827	4.12839	94	235.4	0.008814793	0.52339
45	283.7	0.005359669	4.43158	95	235.1	0.070969258	73.65438
46	282.4	0.016791566	1.8996	96	233.9	0.004545903	6.11684
47	282.1	0.009537766	-0.93752	97	233.8	0.045336944	-83.81153
48	279.2	0.078404663	-82.75387	98	233.7	0.001330075	0.12432
49	278.7	0.010245449	42.55841	99	233.4	0.002664690	-4.39639
50	276.7	0.019391761	-22.74536	100	232.6	0.027123877	-17.82919

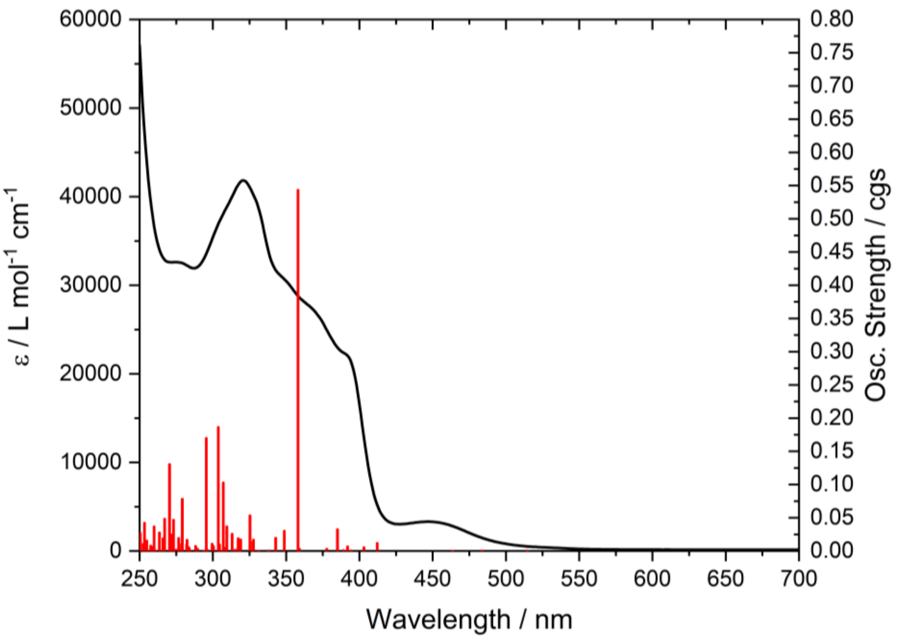

Figure S23. Experimental UV-Vis spectrum of compound *rac*-$[Cr(dqp)(ddpd)]^{3+}$ in CH_3CN and calculated oscillator strength of the calculated electronic transitions.

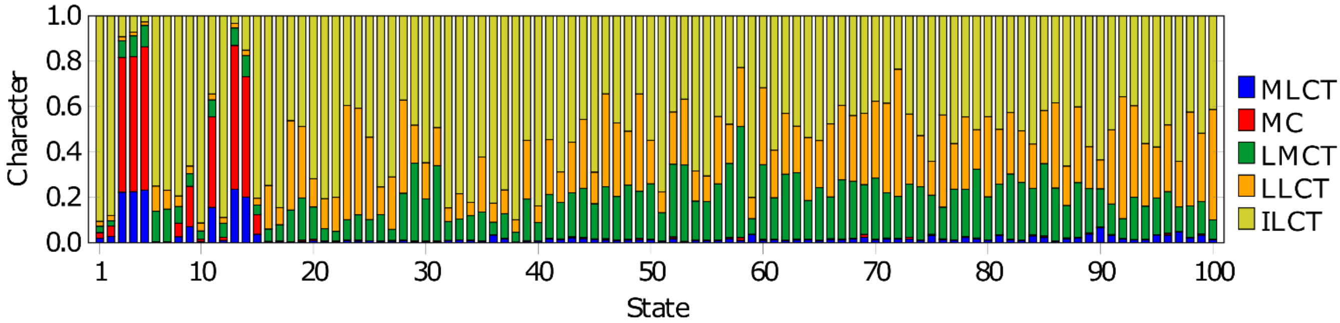

Figure S24. TD-DFT charge transfer numbers of $[Cr(dqp)(ddpd)]^{3+}$ defined from 0 to 1 of the first 100 electronic transitions.

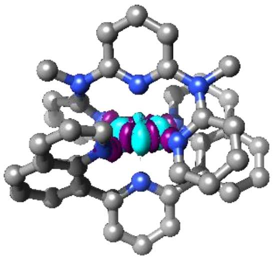

#3 – 486.9 nm – MC
$d_{xy} \rightarrow d_{x^2-y^2}$

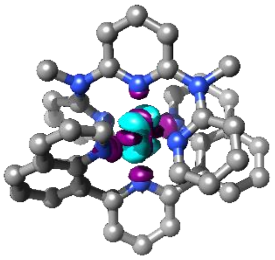

#4 – 483.5 nm – MC

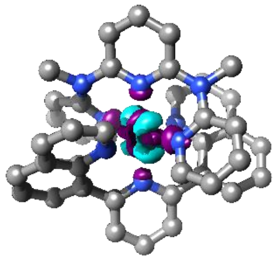

#5 – 463.5 nm – MC

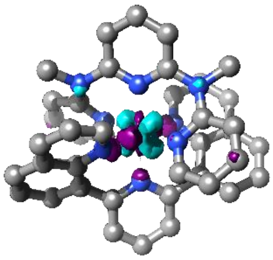

#11 – 389.1 nm – MC

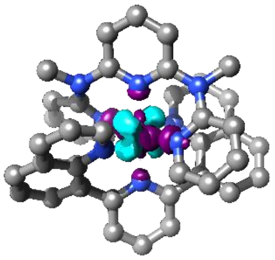

#13 – 377.7 – MC

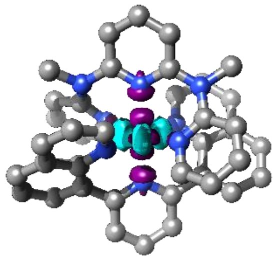

#14 – 372.4 nm – MC
$d_{xy} \rightarrow d_{z^2}$

Figure S25. Electron density difference maps (EDDMs) for the metal-centered transitions of $[Cr(dqp)(ddpd)]^{3+}$. Blue: density loss; Purple: density gain.

Table S12. Excited state lifetime, luminescence quantum yield and radiative rate (k_{rad}) values of the $Cr(^2T(1)\rightarrow{}^4A_2)$ transition for the $[Cr(dqp)_2]^{3+}$, $[Cr(ddpd)_2]^{3+}$, $[Cr(dqp)(ddpd)]^{3+}$ and $[Cr(qpp)_2]^{3+}$complexes in aerated acetonitrile solution at 293 K.

Complex	τ_{obs} (s^{-1})	Φ	k_{rad}
$[Cr(dqp)_2]^{3+}$	$80{\cdot}10^{-5}$	0.007	87
$[Cr(dqp)(ddpd)]^{3+}$	$14{\cdot}10^{-5}$	0.0013	93
$[Cr(qpp)_2]^{3+}$	$33{\cdot}10^{-5}$	0.004	121
$[Cr(ddpd)_2]^{3+}$	$51{\cdot}10^{-5}$	0.0064	125

Geometries

HH-$[Cr(qpp)_2]^{3+}$

Cr	4.00493698872780	8.79512354289060	13.40796862137545
N	3.65947482092644	8.13215089523684	11.46475900492275
N	2.18855050812676	9.73277333394869	13.34053158425844
N	3.11057924408325	11.50795210020766	14.62778665554343
N	4.18610974669266	9.53105220583055	15.31636883344770
N	3.16855331809294	7.07927772824702	14.23747672512987
N	5.82511760824151	7.86450133164171	13.48224887093098
N	6.82711747905018	9.95844272482433	14.00437085620542
N	4.99090472253958	10.40407807670009	12.60054828275782
C	4.68311409131456	7.92402641338815	10.64304249026359
H	5.59659050871476	8.45725420357342	10.85762683520609
C	4.63388446785603	7.02137499802734	9.58038999475825
H	5.49890490009476	6.90089831348201	8.94652821846703
C	3.51080981273666	6.24956283245803	9.43190646255005
H	3.46378436191067	5.46954988716292	8.68380670709567
C	2.39202980922116	6.46913850420367	10.26096841286908
C	1.22046791381623	5.68795241189415	10.15973598869992
H	1.19410590744872	4.87927793042182	9.44268960391618
C	0.14999416776554	5.95720546144542	10.96929777173583
H	-0.73894145253999	5.34460187232564	10.92948998565963
C	0.17981134610147	7.06850134670581	11.82576185168612
H	-0.70428911724983	7.29971668805412	12.40113681811195
C	1.29371493089584	7.88199984708518	11.94653592952400
C	2.46364150650079	7.51049046500439	11.22742372357891
C	1.15204940784454	9.17607982366876	12.65507465620846
C	-0.05294876704860	9.85374330348284	12.53695613550878
H	-0.85069665832323	9.43299763264573	11.94767975580537
C	-0.21036541513346	11.08998398382014	13.14377579986233

H	-1.15019559775318	11.61837776911756	13.06835143991656
C	0.83854219924216	11.64153851495342	13.84625197294431
H	0.72936570682965	12.58975787228198	14.34502978902208
C	2.03838135260367	10.93743023629804	13.93293806172534
C	3.17580808775729	12.97655928398851	14.66902704974042
H	2.55969178642332	13.40314493755874	15.46082112047007
H	2.84837338432972	13.35981536224312	13.70822978850345
H	4.21218850457995	13.26464007778223	14.81933404998141
C	3.85152356808539	10.80849421973350	15.57330400504349
C	4.25371279287296	11.42422247120315	16.76439314755207
H	3.99086527945670	12.44968168221901	16.96114664835387
C	4.95157384069289	10.69121378166867	17.70019733101628
H	5.25631771837332	11.15694595638955	18.62687458730021
C	5.23172671144260	9.34741663555266	17.45620622938159
H	5.75821645743118	8.73607532213013	18.17268204294465
C	4.83069612463260	8.80889993994136	16.26002952265937
H	5.03946256814869	7.78038398421497	16.01471285835460
C	2.08963666558937	7.17031067045178	15.00785787444854
H	1.91528931779007	8.11986949016952	15.49016668951115
C	1.17414277166519	6.12821514859087	15.16051160628725
H	0.31850841187482	6.26367593082498	15.80404197227917
C	1.34746586396304	4.99312298505855	14.41164989146479
H	0.61248852014386	4.19951529019477	14.42152055401667
C	2.50122670925007	4.84556114835588	13.61510437257049
C	2.71799318436123	3.70296207694509	12.81467286154400
H	1.95957829734613	2.93291039023557	12.78995216849572
C	3.86927650628539	3.59282199612319	12.08206917729531
H	4.03251102156268	2.74180776623016	11.43686294987461
C	4.87465745277103	4.56418705348177	12.20461898387339
H	5.80282389964053	4.41683265173441	11.67255729136908

C	4.72258143283092	5.69297701135059	12.99185957443646
C	3.46600480964109	5.89053313833232	13.62766951767359
C	5.91690130011054	6.52071246936391	13.28301419480599
C	7.13503300418759	5.87294117157131	13.42797525948450
H	7.18944304856082	4.80222981117352	13.32199403511057
C	8.26828267223864	6.60235651533758	13.75215536684580
H	9.22336677756887	6.10717807695371	13.85575679406292
C	8.17086753818758	7.96363622175522	13.93892705419206
H	9.04365365215502	8.55167513021380	14.16703866769981
C	6.92851326938711	8.57912654846063	13.79425351727245
C	7.79379697034470	10.56708459754239	14.93141674494154
H	7.95666094359392	9.87772698815021	15.75331214829980
H	7.35505069241391	11.47750686514462	15.32935890639938
H	8.74625849364609	10.79927822105546	14.45440232721119
C	6.16225000012894	10.80586076723782	13.12568867729630
C	6.69504984145967	12.05905141898274	12.80154949679152
H	7.62942479455251	12.37903063155570	13.23042785184718
C	6.03428723899393	12.85885839839077	11.89397770624677
H	6.44626280069896	13.82265061581922	11.62958626141099
C	4.85683198117248	12.40423958854874	11.30155043680616
H	4.32210951144862	12.99251154751403	10.57198345162934
C	4.37430217871462	11.17700324470049	11.67864197440596
H	3.46071178216318	10.78473251679455	11.26265742110992

$[Cr(dqp)(ddpd)]^{3+}$

Cr	4.84993434262922	14.32545717367829	9.86602763078945
N	4.87795783051339	16.39009639521563	9.66797612608751
N	6.88899951455298	14.37466569045957	9.95017302719209
N	4.96660064947230	12.26467422731657	10.07878790966138
N	4.64905685463747	14.43499533164193	11.90930523036920
N	2.80306152125033	14.26650054902182	9.77586557948373
N	4.83386036121736	14.21467366727517	7.81308057388148
N	2.70178123308809	15.73623384544038	11.65042507346397
N	2.95649993635443	12.79579341114638	7.90455618395196
C	3.92600643304819	17.00698144431479	8.97666661166303
H	3.39703505416988	16.41791497738240	8.24348873740856
C	3.56401026769584	18.33592478277921	9.20108181003466
H	2.78819818508044	18.78329243700329	8.59891493683856
C	4.15224189666179	19.00611241044871	10.24258918341210
H	3.83762817543140	20.00514628246532	10.51326832585123
C	5.18056216589965	18.38956253618698	10.98332849644839
C	5.60047845230003	17.08694833445861	10.59690289308008
C	5.78980311755731	19.02102396778939	12.09035392590235
H	5.43850488847251	19.99889537628546	12.38974498962780
C	6.79580435056249	18.38621772152903	12.76758728149198
H	7.24327252570155	18.83883652411963	13.64061590927036
C	7.29835088658305	17.16426193579592	12.29340162350192
H	8.14419193471898	16.72385455486980	12.79990510861418
C	6.76020752785559	16.51998664707606	11.19282561318911
C	7.51227658811010	15.39613927552448	10.58933631821958
C	8.90312488901015	15.44692730543739	10.64194987332233
H	9.39069102118968	16.28725220825988	11.10776119929555
C	9.65044240148167	14.43561166756220	10.07108132553020

H	10.72993791363990	14.45903908312507	10.11928254748180
C	9.00096767013868	13.39388797477950	9.43797300167420
H	9.56341837748124	12.57585129787038	9.01951632397739
C	7.61033211497653	13.38353377516325	9.36830949795184
C	6.96404281685715	12.22802798186845	8.70477326318784
C	7.61611491964533	11.61322972560243	7.65000940228098
H	8.47736712443607	12.09372846174663	7.20962852974836
C	7.20941935443735	10.37003823046133	7.13941929289784
H	7.74372679309376	9.94169797702821	6.30378497667140
C	6.18515145945831	9.68643566548988	7.73731237331290
H	5.90413055997914	8.69430931740821	7.41170983150913
C	5.46391485500923	10.28443270395006	8.79432654571640
C	5.79068086785326	11.60436673684408	9.21022130375807
C	4.41158195740730	9.61843374977553	9.45448205727070
H	4.16565538812073	8.60656733618197	9.16132846429210
C	3.71402434358066	10.25813527179342	10.44638446157108
H	2.91566278732743	9.77373013013163	10.98731956748434
C	3.99375443955766	11.60242801762345	10.69501234452564
H	3.37950050307976	12.16449445432531	11.38135849336458
C	5.58659612629098	13.86219247982224	12.69729952451522
H	6.28033778026328	13.20095389226234	12.20475326665339
C	5.68085375960684	14.11925850395748	14.04113784304294
H	6.45720862627365	13.65126201184824	14.62642610934993
C	4.76035498078191	14.99959290817392	14.60680229098074
H	4.80169695034295	15.23702216011950	15.66060942818791
C	3.76706155326634	15.54793690706732	13.82347624239946
H	3.02285605932046	16.18842569367899	14.26426907993602
C	3.71271381296838	15.22871912704694	12.46166756176916
C	1.96020938553786	16.90176233282061	12.15790391151712
H	1.23264512526594	16.63353150361483	12.92426368227680

H	1.45243642313028	17.37291885108215	11.32340989651442
H	2.67341368524753	17.60772815755147	12.57126621230553
C	2.05967316366478	14.96458832267423	10.66952474097990
C	0.67168907019089	14.94397339172676	10.62231841328300
H	0.09215307067929	15.47921175098989	11.35452069683445
C	0.04621551760956	14.19469895951094	9.64410231249170
H	-1.03300643132210	14.16692323360602	9.59216762915791
C	0.80009781886086	13.48007785307335	8.73271932147937
H	0.32265290532068	12.91657628395334	7.94972245369329
C	2.18505362459266	13.53195834766163	8.81815657944768
C	2.32674543461488	11.59383658649643	7.33573828299684
H	1.76984036949930	11.09583955443639	8.12183016162527
H	3.11054016650776	10.92669162391526	6.99191979531402
H	1.65944200858934	11.82486772847536	6.50505030268363
C	4.00425495800845	13.36165204955913	7.18394648614866
C	4.20542799441307	13.04132640477942	5.83610249194661
H	3.55007662227951	12.35022161664950	5.33475408247627
C	5.22892701146824	13.65179924327598	5.14254227786894
H	5.38360542796429	13.41356215437749	4.09957286503967
C	6.03315190012514	14.59331035953830	5.78192093119059
H	6.82764857590929	15.10961403212620	5.26570414823462
C	5.80020000854529	14.84639901934816	7.10952899520103
H	6.40375926285670	15.55131838312609	7.65751820619790
